# Aberrant neurodevelopment in human iPS cell‐derived models of Alexander disease

**DOI:** 10.1002/glia.24618

**Published:** 2024-09-23

**Authors:** Zuzana Matusova, Werner Dykstra, Yolanda de Pablo, Oskar G. Zetterdahl, Isaac Canals, Charlotte A. G. H. van Gelder, Harmjan R. Vos, Dolores Pérez‐Sala, Mikael Kubista, Pavel Abaffy, Henrik Ahlenius, Lukas Valihrach, Elly M. Hol, Milos Pekny

**Affiliations:** ^1^ Laboratory of Gene Expression Institute of Biotechnology of the Czech Academy of Sciences Vestec Czechia; ^2^ Faculty of Science Charles University Prague Czechia; ^3^ Department of Translational Neuroscience University Medical Centre Utrecht Brain Centre, Utrecht University Utrecht The Netherlands; ^4^ Laboratory of Astrocyte Biology and CNS Regeneration, Center for Brain Repair, Department of Clinical Neuroscience Institute of Neuroscience and Physiology, Sahlgrenska Academy at the University of Gothenburg Gothenburg Sweden; ^5^ Stem Cells, Aging and Neurodegeneration Lab, Department of Experimental Medical Science, Faculty of Medicine, Lund Stem Cell Center Lund University Lund Sweden; ^6^ Glial and Neuronal Biology Lab, Department of Experimental Medical Science, Faculty of Medicine, Lund Stem Cell Center Lund University Lund Sweden; ^7^ Division of Metabolism University Children's Hospital Zurich, University of Zurich Zurich Switzerland; ^8^ ITINERARE–Innovative therapies in rare diseases, University Research Priority Program, University of Zurich Zurich Switzerland; ^9^ Oncode Institute and Molecular Cancer Research, Center for Molecular Medicine University Medical Center Utrecht, Utrecht University Utrecht The Netherlands; ^10^ Centro de Investigaciones Biológicas Margarita Salas Madrid Spain; ^11^ Institute of Biomedicine, University of Gothenburg Gothenburg Sweden; ^12^ Department of Cellular Neurophysiology Institute of Experimental Medicine of the Czech Academy of Sciences Prague Czechia; ^13^ Florey Institute of Neuroscience and Mental Health Parkville Victoria Australia; ^14^ University of Newcastle Newcastle New South Wales Australia

**Keywords:** Alexander disease, GFAP, iPS cells, neural organoids

## Abstract

Alexander disease (AxD) is a rare and severe neurodegenerative disorder caused by mutations in glial fibrillary acidic protein (GFAP). While the exact disease mechanism remains unknown, previous studies suggest that mutant GFAP influences many cellular processes, including cytoskeleton stability, mechanosensing, metabolism, and proteasome function. While most studies have primarily focused on GFAP‐expressing astrocytes, GFAP is also expressed by radial glia and neural progenitor cells, prompting questions about the impact of GFAP mutations on central nervous system (CNS) development. In this study, we observed impaired differentiation of astrocytes and neurons in co‐cultures of astrocytes and neurons, as well as in neural organoids, both generated from AxD patient‐derived induced pluripotent stem (iPS) cells with a GFAP^R239C^ mutation. Leveraging single‐cell RNA sequencing (scRNA‐seq), we identified distinct cell populations and transcriptomic differences between the mutant GFAP cultures and a corrected isogenic control. These findings were supported by results obtained with immunocytochemistry and proteomics. In co‐cultures, the GFAP^R239C^ mutation resulted in an increased abundance of immature cells, while in unguided neural organoids and cortical organoids, we observed altered lineage commitment and reduced abundance of astrocytes. Gene expression analysis revealed increased stress susceptibility, cytoskeletal abnormalities, and altered extracellular matrix and cell–cell communication patterns in the AxD cultures, which also exhibited higher cell death after stress. Overall, our results point to altered cell differentiation in AxD patient‐derived iPS‐cell models, opening new avenues for AxD research.

## Main Points

- Astrocytes, neurons and neural organoids from AxD patient‐derived iPS cells show impaired neural differentiation and altered lineage commitment.
- Neuron/astrocyte co‐cultures and neural organoids from AxD iPS cells are more susceptible to stress.

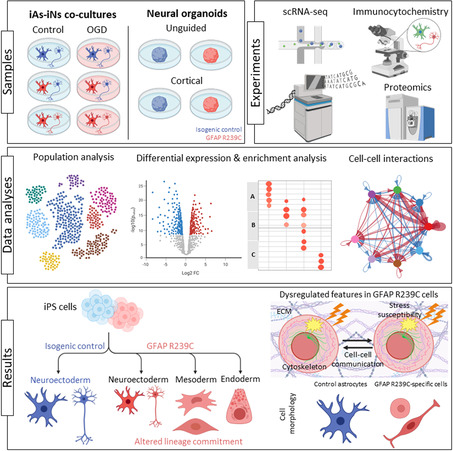

## 1 INTRODUCTION

Alexander disease (AxD) is a rare and severe neurodegenerative disorder affecting primarily the white matter. It is caused by mutations in glial fibrillary acidic protein (GFAP), which in the central nervous system (CNS) is expressed by astrocytes and neural stem cells. Mutant GFAP together with several associated proteins form aggregates in astrocytes known as Rosenthal fibers (RFs), which are the main neuropathological hallmark of AxD (reviewed in Hagemann (2022), Pajares et al. (2023), Pekny et al. (2016)). To date, a number of pivotal studies using rodent (Hagemann et al., 2005; Hagemann et al., 2006; Heaven et al., 2022; Meisingset et al., 2010; Tanaka et al., 2007), *Drosophila* (Wang et al., 2011), zebrafish (Lee et al., 2017), human cell culture models (Jones et al., 2018; Kondo et al., 2016), or patient post‐mortem brain samples (Olabarria et al., 2015; Tang et al., 2010; Walker et al., 2014) brought important insights into AxD pathogenesis, revealing that overexpression and aggregation of mutant GFAP in astrocytes is accompanied by a stress response, neuroinflammation, and reactive gliosis as well as disruption of vesicular trafficking, proteasome function, and glutamate and calcium homeostasis. AxD astrocytes exhibit altered posttranslational modifications of GFAP (Battaglia et al., 2019; Viedma‐Poyatos et al., 2022), impaired intermediate filament organization (Yang et al., 2022) and their increased sensitivity to oxidative stress further enhances GFAP aggregation (Viedma‐Poyatos et al., 2022). Sosunov et al. observed AxD astrocytes with reactive‐like features, some of them containing multiple nuclei as a consequence of mitosis arrest (Sosunov et al., 2013; Sosunov et al., 2017). Abnormal organelle morphology and distribution (Jones et al., 2018), including mitochondria (Viedma‐Poyatos et al., 2022), were detected in AxD astrocytes. Impaired mitochondria transfer between astrocytes and neurons may contribute to the disease pathogenesis (Gao et al., 2019). Linking GFAP mutation to white matter degeneration, AxD astrocytes were shown to inhibit proliferation of oligodendrocyte progenitor cells (OPCs) and reduce their myelination potential (Li, Tian, et al., 2018).

GFAP is also expressed in multipotent neural stem cells (Imura et al., 2003) and in radial glia (RG) of the developing brain (Dimou & Gotz, 2014), but the effect of GFAP mutations on brain development is largely unexplored. Hagemann et al. (2013) reported aberrant adult neurogenesis in the hippocampus of the Gfap^+/R236H^ AxD mouse model, observing RG‐like cells with RFs and hypertrophic morphology, absence of immature neurons, and diminished proliferation of neural progenitors, possibly a consequence of disturbed ubiquitin‐proteasome system, which normally regulates essential developmental pathways such as Notch, WNT, Hedgehog, and TGFβ (Baloghova et al., 2019; Dutta et al., 2022; Gao et al., 2014; Hsia et al., 2015; Imamura et al., 2013).

Here, we used an AxD patient‐derived induced pluripotent stem (iPS) cell line carrying a GFAP^R239C^ mutation and a CRISPR/Cas9‐corrected isogenic control cell line to generate astrocyte‐neuron co‐cultures, as well as unguided neural organoids and cortical organoids. Leveraging single‐cell RNA sequencing (scRNA‐seq), we describe a differentiation impairment and increased sensitivity to oxygen–glucose deprivation (OGD) challenge in AxD co‐cultures. In AxD unguided neural organoids and cortical organoids, we observed almost complete absence of astrocytes, reduced neurogenesis and enrichment of mesoderm‐ and endoderm‐derived cell populations, suggesting a neural lineage commitment defect.

## 2 METHODS

### 2.1 iPS cells

AxD iPS cells, derived from a 6‐year‐old AxD patient carrying the heterozygous point mutation in *GFAP* (c.715C > T, p.R239C), and isogenic CRISPR/Cas9‐corrected control iPS cells were received from Natasha Snider (Department of Cell Biology and Physiology, University of North Carolina at Chapel Hill, USA) and were described previously (Battaglia et al., 2019).

### 2.2 Lentiviral production

Lentiviral vectors used were M2‐rtTA (rtTA, reverse tetracycline‐controlled transactivator, Addgene #20342), tet‐O‐Ngn2‐puro (Addgene #52047, Zhang et al. (2013)), tetO‐Sox9‐Puro (Addgene #117269), and tetO‐Nfib‐Hygro (Addgene #117271). Nfib, Ngn2, Sox9, and rtTA lentiviruses were produced in HEK 293 T cells, as described in Canals et al. (2018). Briefly, the HEK 293 T cells were cotransfected with the packaging plasmids pMDLg/pRRE (Addgene #12251), pMD2.G (Addgene #12259), and pRSV‐Rev (Addgene #12253) in addition to the lentivectors, approximately 44 h following transfection the viruses were pelleted by centrifugation (20,000 rpm at 4°C for 2 h), and the supernatant was subsequently aspirated. A total of 100 μL of Dulbecco's modified Eagle's medium (DMEM) was added to the pellet without resuspending. One day later, the viruses were resuspended, aliquoted and frozen at −80°C for long‐term storage.

### 2.3 iPS cell culturing for neuronal and astrocyte co‐cultures

Cell lines were maintained in mTesR1/mTesR+ media on ES‐qualified Matrigel‐coated 6 W plates at 37°C in humidified air with 5% CO_2_ with daily medium change. Cells were passaged with accutase (StemPro Accutase Cell Dissociation Reagent) upon attaining confluency of ~80%. Upon dissociation, the cells were centrifuged at 300 × *g* and replated onto fresh Matrigel‐coated 6‐well plates at a density of 2–2.5 × 10^5^ cells/well using medium supplemented with 10 μM of ROCK Inhibitor (RI) in the initial 24 h after replating to enhance the rate of cell survival.

### 2.4 Differentiation of induced astrocytes

Human iPS cells at ~80% confluency were dissociated with accutase on Day −2, and 4 × 10^5^ cells were plated on Matrigel‐coated 6‐well plates with mTeSR supplemented with 10 μM RI. The following day (Day −1), the medium was replaced with fresh mTeSR medium, and 1 μL of rtTA Sox9 and Nfib lentivirus was added to each well. On Day 0, the medium was replaced with fresh mTeSR medium containing Doxycycline (Dox; 2.5 μg/mL), which was added to the medium throughout the experiments. Sox9 and Nfib lentiviral overexpression was used for astrocyte induction (Canals et al., 2018). On Days 1 and 2 the iAs were cultured in expansion medium (DMEM/F12, 10% FBS, 1% N2, and 1% GlutaMax). Between Day 3 and 5, expansion medium was gradually changed to FGF medium (Neurobasal, 2% B27, 1% NEEA, 1% GlutaMax, 1% FBS, 8 ng/mL FGF, 5 ng/mL CNTF, and 10 ng/mL BMP4). Overall, 72 h of puromycin (2.5 μg/mL) selection and 5 days of hygromycin (200 μg/mL) selection was performed.

### 2.5 Differentiation of glutamatergic induced neurons

Human iPS cells at ~80% confluency were dissociated with accutase on Day −2, and 3 × 10^5^ cells were plated on Matrigel‐coated 6‐well plates with mTeSR‐1 supplemented with 10 μM RI. The following day (Day −1), the medium was replaced with fresh mTeSR‐1 medium, and 1 μL of rtTA and Ngn2 lentivirus was added to each well. On Day 0, the medium was replaced with fresh mTeSR‐1 medium containing Doxycycline (Dox; 2.5 μg/mL), which was added to the medium throughout the experiments. Ngn2 lentiviral overexpression was used for neuronal induction (Zhang et al., 2013). From Day 1, supplemented BrainPhys medium (BrainPhys, 0.5% N2 supplement, 1% B27 supplement) was used and 72 h of puromycin (2.5 μg/mL) selection was performed. From Day 5, the medium was supplemented with NT3 (10 ng/mL) and BDNF (10 ng/mL).

### 2.6 Co‐cultures of iAs and iNs

On Day 7, iAs and iNs were dissociated with accutase supplemented with DNase I for 10 min. The iAs were then pelleted for 5 min at 300 × *g*. The iNs were dissociated in accutase for 10 min, followed by manual resuspension in the accutase for 5 more minutes before being pelleted for 5 min at 300 × *g*. The iNs were then strained through a 40 μm mesh to avoid neuronal aggregates. 3.9 × 10^4^ iAs were plated together with 1.11 × 10^5^ iNs on PEI + rhLam521‐coated Ibidi 24 well μ‐Plates. For the scRNAseq, 2.6 × 10^5^ iAs and 7.4 × 10^5^ iNs were plated on Matrigel‐coated six well plates. The medium henceforth consisted of 50% iNs medium and 50% iAs medium. On Day 8, the medium was replaced with 1:1 BrainPhys and FGF medium. From Day 9 on, the FGF medium was changed to Maturation medium (1:1 DMEM/F12 and Neurobasal, 1% N2, 1% Na Pyruvate, 10 μg/mL NAC, 10 ng/mL hbEGF, 10 ng/mL CNTF, 10 ng/mL BMP4, 500 μg/mL dbcAMP) and half of the medium would be changed every 2 days. Between Days 9 and 21, 5‐Fluoro‐2′‐deoxyuridine (FUDR; 20 μM/mL) was added to the medium to inhibit cell division. Co‐cultures were maintained for 42 days before being used for experiments.

### 2.7 Oxygen–glucose deprivation challenge

After 5 weeks in co‐culture, medium was changed to deoxygenated ischemic medium (DMEM w/o glucose, 2.5 μg/mL Dox, 5 ng/mL NT3, 5 ng/mL BDNF, 10 μg/mL NAC, 10 ng/mL hbEGF, 5 ng/mL CNTF, 5 ng/mL BMP4, 250 μg/mL dbcAMP) for cells to be subjected to the OGD, and co‐culture medium for the control (1:1 BrainPhys and Maturation media, with factors). The OGD cells were incubated at 37°C in humidified air with 1% O_2_ and 5% CO_2_, and control cells at normal culturing conditions (37°C in humidified air with 5% CO_2_), for 4 h. Subsequently, a medium change to fresh co‐culture media for all cells was performed, followed by 2 h of recovery under normal culturing conditions for the LDH assay and 16 h of recovery for the single‐cell RNA sequencing.

### 2.8 LDH assay

Media samples were taken following the 2 h reperfusion period after OGD challenge, from both OGD‐exposed and control cells. One well of each condition was lysed by adding Triton‐X 100 to final concentration of 1%, to obtain the Max LDH release. Cytotoxicity was assessed using Takara Bio LDH Cytotoxicity Detection Kit. Absorbance was measured at 490 nm (600 nm as a reference wavelength) and cytotoxicity was calculated using the formula: Cytotoxicity (%) = (Absorbance_(490–600)_ LDH Sample/Absorbance_(490–600)_ Max LDH Release) × 100.

### 2.9 Single‐cell suspension for scRNA‐seq

Medium was aspirated and cells were washed with cold phosphate‐buffered saline (PBS)^−Ca/−Mg^. Cells were dissociated with accutase supplemented with 30 μM ActD and incubated until most cells were detached. The accutase was then inhibited with DMEM supplemented with 3 μM ActD and 2% B27 and cells were collected in LoBind 15 mL tubes and kept on ice for the rest of the protocol. Using a wide‐bore pipette, cells were gently resuspended. Cells were strained before being centrifuged at 250 × *g* at 4°C for 5 min. Supernatant was removed and cells were gently resuspended in cold DMEM (with 3 μM ActD and 2% B27) using a wide‐bore pipette tip. Cells were centrifuged at 150 × *g* at 4°C for 5 min. Supernatant was once again removed. The volume of cell suspension was adjusted using cold DMEM with 3 μM ActD and 2% B27. Samples were then strained, and an additional cell count was performed. If necessary, the volumes of samples were adjusted to obtain a target cell concentration of 700–1200 cells/μL and a target cell count of 10,000 cells per sample.

### 2.10 Immunofluorescence of co‐cultures

For immunofluorescence imaging, cells were plated on PEI + rhLam521‐coated Ibidi 24‐well μ‐Plates. Cells were washed with room temperature PBS ^Ca/Mg^ and then fixed in 4% paraformaldehyde (PFA) in PBS at room temperature for 15 min. Cells were washed three times with potassium‐phosphate‐buffered saline (KPBS) before being blocked with KPBS containing 0.025% Triton X‐100 (TKPBS) and either 5% normal donkey serum or 2.5% normal donkey serum and 2.5% normal goat serum for 60 min. Primary antibodies were incubated in blocking solution overnight at 4°C. Cells were washed twice with 0.025% TKPBS and once with blocking solution for 5 min. Secondary antibody together with nuclear staining incubation was performed at room temperature for 2 h in blocking solution. Cells were washed once with 0.025% TKPBS for 5 min and twice for 5 min with KPBS, PVA:DABCO was used to mount coverslips on top of stained cells.

Images of the co‐cultures were acquired using an epifluorescence Leica microscope and were analyzed using Fiji (Schindelin et al., 2012). Images for the assessment of the morphology of GFAP‐positive cells were acquired using a 40× objective. The GFAP outline was traced manually, circularity, and perimeter were measured using Fiji shape descriptors. Each datapoint represents a cell. Images for cell counts were taken using a 10× objective, MAP2^+^, Vimentin^+^, or GFAP^+^ cells were counted manually. Undifferentiated cells were identified manually based on their spherical morphology on phase contrast images. Each datapoint represents a separate culture. Normal distribution of the data was tested with the Shapiro–Wilk normality test. The Wilcoxon rank sum test with continuity correction was used to compare nonnormally distributed data, Student's *t*‐test was used to compare normally distributed data.

### 2.11 iPS cell culture for neural organoid generation

iPS cells were maintained in Stemflex medium (Life technologies, A3349401) on Geltrex‐coated (Life technologies, A1413202) dishes in feeder‐free conditions at 37°C with 5% CO_2_. The medium was changed daily, and cells were split in Stemflex medium containing 5 μM Y27632 (Axon Biochemicals, AXON 1683) as soon as they reached 80% confluency by incubating them with 0.5 mM EDTA for 3 min at 37°C. After 24 h, the medium was changed to regular Stemflex medium. The number of passages was kept below 40 and routine testing for mycoplasma (Lonza, LT07‐318, Lonza Bioscience Solutions, Basel, Switzerland) was performed.

### 2.12 Generation of unguided neural organoids

Unguided neural organoids were generated according to a combined protocol of Ormel et al. (2018), Lancaster et al. (2013), and Verkerke et al. (2024). Briefly, at Day 0, iPS cells that reached 80% confluency were dissociated into single cells following a 2‐min incubation period with 0.5 mM EDTA in PBS and a 4‐min incubation period with accutase at 37°C. After counting, ~9000 iPS cells were allowed to form an embryoid body at 37°C with 5% CO_2_ in a well of an ultra‐low attachment round‐bottom shaped 96‐well plate (Corning 3474) containing a total of 150 μL HES medium consisting of DMEM/F‐12 (ThermoFisher Scientific, 31330038), 20% KOSR (Life Technologies,10828028), 3% FBS (ThermoFisher Scientific, 10082147), 1% l‐glutamine (Life Technologies, 25030024), 1 % NEAAs (ThermoFisher Scientific, 11140035), 0.1 mM 2‐Mercaptoethanol (Merck, 8057400005) supplemented with 4 ng/mL bFGF (Peptro‐Tech 100‐18B), and 50 μM Y27632 (Axon Biochemicals, AXON 1683) (HES4+ medium). At Day 2, 100 μL of medium was replaced by 150 μL of HES4+ medium. At Day 4, 150 μL of medium was removed and replaced by 150 μL HES medium. At Day 6, 150 μL of medium was replaced with 150 μL neural induction medium (NIM) consisting of DMEM/F‐12 (ThermoFisher Scientific, 31330038), 1 % N2 (Life Technologies, 17502048), 1 % L‐glutamine (Life Technologies, 25030024), 1 % NEAA (ThermoFisher Scientific, 11140035), and 0.5 μg/mL heparin (Sigma‐Aldrich, H3149). At Day 8, 10, and 12, 150 μL of the medium was replaced with fresh NIM. At Day 13, unguided neural organoids were embedded in 30 μL droplets of Matrigel (Corning, 356234), incubated for 25 min at 37°C, and transferred to a 6 cm ultra‐low attachment culture dish (Corning, 3261) containing 6 mL of differentiation medium without vitamin A (B27‐medium) consisting of DMEM/F‐12 (ThermoFisher Scientific, 31330038) in a 1:1 ratio with neurobasal medium (ThermoFisher Scientific, 21103049) supplemented with 1% B27‐vitamin A (ThermoFisher Scientific, 12587001), 1 % penicillin–streptomycin (Life Technologies, 15140122), 1% L‐glutamine (Life Technologies, 25030024), 0.5% NEAA (ThermoFisher Scientific, 11140035), 0.5% N2 (Life Technologies, 17502048), 125 μL Insulin (Sigma‐Aldrich, I9278), 174 μL 2‐Mercaptoethanol (Merck, 8057400005) diluted 1:100 in DMEM/F‐12. After 4 days of stationary culture, at Day 17, unguided neural organoids were transferred to an orbital shaker and cultured in 6 mL of differentiation medium containing vitamin A (B27+ medium) consisting of DMEM/F‐12 (ThermoFisher Scientific, 31330038), neurobasal medium (ThermoFisher Scientific, 21103049) (1:1) supplemented with 1% B27 + vitamin A (ThermoFisher Scientific, 17504001), 1% penicillin–streptomycin (Life Technologies, 15140122), 1% L‐glutamine (Life Technologies, 25030024), 0.5% NEAA (ThermoFisher Scientific, 11140035), 0.5% N2 (Life Technologies, 17502048), 125 μL Insulin (Sigma‐Aldrich, I9278), and 174 μL 2‐Mercaptoethanol (Merck, 8057400005) diluted 1:100 in DMEM/F‐12. Until harvested, the medium was replaced every 3 to 4 days.

### 2.13 Generation of cortical organoids

Cortical organoids were generated according to an adapted version of Yoon et al. (2019). Briefly, iPS cells that reached 80% confluency were dissociated into single cells following a 7‐min incubation period with accutase at 37°C. After counting, 3.5 × 10^6^ iPS cells were seeded in a well of an Aggrewell™800 plate (STEMCELL technologies, 34811) in 2 mL human embryonic stem (HES) medium consisting of DMEM/F‐12 (ThermoFisher Scientific, 31330038), 20% KOSR (Life Technologies, 10828028), 3% FBS (ThermoFisher Scientific, 10082147), 1% Glutamax (ThermoFisher Scientific, 35050061), 1% NEAAs (ThermoFisher Scientific, 11140035), 3.5 μL 2‐Mercaptoethanol (Merck, 8057400005) supplemented with 4 ng/mL bFGF, and 50 μM Y27632 (Axon Biochemicals, AXON 1683) (HES4+). Embryoid bodies were allowed to form at 37°C with 5% CO_2_. After 24 h, 1.5 mL of medium was replaced with fresh HES4+ medium. At Day 2, properly formed embryoid bodies were transferred to an ultra‐low attachment round‐bottom shaped 96‐well plate (Corning, 3474) containing a total of 150 μL of HES medium supplemented with two SMAD pathway inhibitors—Dorsomorphin (2.5 μM; Tocris 3093) and +SB431542 (10 μM; Axon biochemicals 1661) to start neural induction. At Day 4, 100 μL of medium was replaced with 150 μL of fresh medium. At Day 6, 150 μL of medium was replaced by 150 μL of neural medium consisting of neurobasal medium (ThermoFisher Scientific, 21103049), 2% B27‐vitamin A (ThermoFisher Scientific, 12587001), 1% penicillin–streptomycin (Life Technologies, 15140122), and 1% L‐glutamine (Life Technologies, 25030024) supplemented with 20 ng/mL EGF (R&D systems 236‐EG) and 20 ng/mL bFGF (Peptro‐Tech 100‐18B). After the medium was refreshed every 2 days, at Day 24, EGF and bFGF were replaced by 20 ng/mL BDNF (STEMCELL Technologies 78005.1) and 20 ng/mL NT‐3 (Tebu‐bio 450‐03‐B). After a period of bi‐daily medium changes, at Day 42, cortical organoids were transferred to a 6 cm ultra‐low attachment culture dish (Corning, 3261), and the medium was replaced by neural medium without the addition of growth factors. Subsequently, cortical organoids were cultured on an orbital shaker until harvest with medium changes every 3 to 4 days.

### 2.14 Immunofluorescence of organoids

Neural organoids were fixed in 4% PFA in PBS overnight at 4°C and washed three times for 10 min in PBS before being incubated overnight at 4°C in 30% sucrose in PBS. Subsequently, neural organoids were embedded in Tissue‐Tek(R) O.C.T. Compound (Sakura Finetek, 4583), snap‐frozen in a dry ice/ethanol slurry and stored at −80°C until further use. Sections of 20 μm thickness were obtained with a Leica CM1950 cryostat (Leica Biosystems, Illinois, USA), collected on SuperFrost(R) PLUS (VWR, 631‐0108) slides and stored at −80°C until further processing. Sections were blocked in blocking buffer consisting of 10% normal donkey serum (Jackson ImmunoResearch, 017‐000‐121), 3% BSA (Sigma‐Aldrich, A4503‐100) and 0.1% Triton‐X (Sigma‐Aldrich, T8787‐100) in PBS at room temperature for 1 h. Next, samples were incubated with primary antibodies in blocking buffer at 4°C overnight. Slides were then washed three times for 10 min in PBS containing 0.05% Tween 20 (Merck, 817,072) (PBS‐T) before being incubated with secondary antibodies and Hoechst (Sigma‐Aldrich, 94403) in blocking buffer at room temperature for 1 h. Then, slides were washed three times for 10 min in PBS‐T. The antibodies that were used in the current study can be found in Table S2. Finally, samples were mounted on glass coverslips using Fluorosave (CalBioChem, 345789) and imaged using a Zeiss Axioscope A1 (Zeiss, Oberkochen, Germany) and a Zeiss LSM 880 confocal microscope (Zeiss, Oberkochen, Germany). Negative controls are shown in Figure S9.

### 2.15 Quantification of immunofluorescent images of organoids

20× epifluorescent microscopy images were used. To quantify Hoechst^+^, SOX9^+^, and FOXG1^+^ cells, images were processed using ImageJ software as follows. First, background was subtracted using the subtract background function. Next, a threshold was set to generate binary images. To decrease noise, the despeckle function was used. Hereafter, the watershed function was applied to reduce nuclei clumping. Following this, cells were counted using the analyze particles function, whereby the minimum size of the particles was set at 10 μm. Three pictures from the outer edge of three organoids were analyzed, thereby generating nine datapoints. DCX integrated density was measured from four organoids per genotype and presented as integrated density per μm^2^. No difference in the Hoechst signal was observed. Shapiro–Wilk test was used to determine normal distribution of the data and Wilcoxon test (SOX9, FOXG1) and *t*‐test (DCX) were used for statistical comparison between CTRL and AxD samples.

### 2.16 RNA isolation and cDNA synthesis

For RNA isolation, five or more neural organoids were pooled and homogenized in 1 mL of Qiazol (QIAGEN, 79306) with an ULTRA‐TURRAX(R) (IKA, 0003737000), followed by the addition of chloroform in a 1:5 ratio to Qiazol and centrifuged at 12000 × *g* at 4°C for 20 min. The aqueous top phase was collected and mixed with 500 μL isopropanol and stored overnight at −20°C to allow the RNA to precipitate. Subsequently, samples were centrifuged at 12000 × *g* at 4°C for 30 min and the supernatant was aspirated. Pellets were washed three times with 75% ethanol, air‐dried, and dissolved in TE‐buffer (Invitrogen, 12090‐015). RNA concentration was measured using a Varioskan Flash (Thermo Scientific, N06354) or NanoDrop (ThermoFisher Scientific, ND‐2000) and cDNA was synthesized using a Quantitect Reverse Transcription kit (QIAGEN, 205311) as follows. After removal of potential genomic DNA contamination using gDNA wipe‐out buffer from the kit, 500 ng of RNA was reverse transcribed at 42°C for 30 min followed by incubation at 95°C for 3 min to deactivate the RT enzyme. Samples were diluted 1:20 in RNase‐free water and stored at −20°C.

### 2.17 RT‐qPCR

Real‐time quantitative PCR (RT‐qPCR) was performed on QuantStudio 6 Flex Real‐Time PCR System (ThermoFisher Scientific Inc.) using a 384‐well plate under the following conditions: denaturing at 95°C for 10 min, 40 cycles with 95°C for 15 s and annealing at 60°C for 1 min, followed by a dissociation stage where the temperature was increased from 60 to 95°C. Per reaction, 5 μL FastStart Universal SYBR Green Master (Roche, 04913914001), 3 μL MQ (Millipore, SYNS00000), 1 μL cDNA (RNA input concentration 2.5 ng/μL), and 1 μL of 0.5 μmol/mL forward and reverse primer mix. Primers are listed in Table S1. Reactions were run in triplicates. Melting curve analysis was performed as a quality check. Gene expression was normalized to reference genes *SDHA*, *TBP*, and *RPII* in unguided neural organoids and to *GAPDH*, *β‐Actin*, *TBP*, *SDHA*, and *RPII* in cortical organoids, and the data were visualized in a log_2_FC scale of 2^−ΔΔCt^ relative to the average of ΔCt of controls. Normal distribution of the data was assessed with Shapiro–Wilk test. Statistical comparison was made with Student's *t*‐test or the Wilcoxon test.

### 2.18 Preparation of neural organoids for single‐cell RNA sequencing

Eight to 10 neural organoids were washed in an abundance of warm PBS and subsequently chopped into small pieces using a sterile blade. The minced organoids were dissociated in 5 mL of DMEM/F‐12 (ThermoFisher Scientific, 31330038) with freshly dissolved DNAse (1/23 units/mL, Worthington LS006361) and papain (22 units/mL, Worthington LS003118) by gently passing them through a 1 mL pipet tip about 20 times, followed by incubation on an orbital shaker at 37°C with 5% CO_2_ for 10 min. Trituration and centrifugation were repeated three times in total with the last incubation lasting 5 min. After the final dissociation, the enzymes were inactivated by addition of 2% FBS in DMEM/F‐12. Cells were passed through a 40 μm mesh and spun down for 5 min at 300 × *g*. After removal of the supernatant and resuspension in 1 mL of DMEM/F‐12 + 2% FBS, cells were counted using an automated cell counter (Countess II). 1 × 10^6^ cells were fixed for 20 h at 4°C using 10x Fixation of Cells & Nuclei for Chromium Fixed RNA Profiling, (10x Genomics) according to the manufacturer's instructions.

### 2.19 Preparation of scRNA‐seq libraries

Single‐cell suspensions from co‐cultures were processed using Chromium Next GEM Single Cell 3′ Reagent Kit v3.1 (10x Genomics, Pleasanton, CA) according to the manufacturer's instructions by the Center for Translational Genomics, Lund University. Suspension from 165D‐old organoids was fixed and processed using Chromium Fixed RNA Kit (10x Genomics, Pleasanton, CA) according to the manufacturer's instructions. In this case, a pre‐defined set of probes is used to capture protein‐coding genes, mitigating the risk of contamination by ribosomal and mitochondrial genes and other highly abundant transcripts. The concentration and quality of the libraries were measured using Qubit dsDNA HS Assay Kit (Invitrogen) and Fragment Analyzer HS NGS Fragment Kit (#DNF‐474, Agilent).

The libraries were sequenced in paired‐end dual indexing mode with NovaSeq 6000 SP Reagent Kit v1.5 (100 cycles). Cell‐specific 10x GEM Barcodes (16 bp) and molecule‐specific UMIs (12 bp) were contained within Read 1 (28 bp), and target sequence was contained within Read 2 (90 bp). Details can be found in Table S3.

### 2.20 Data processing and analyses

Initial quality control of sequencing data was performed using FastQ Screen (0.11.1, Wingett and Andrews (2018)). Sequences were trimmed with TrimmomaticSE (0.36, Bolger et al. (2014)). STARsolo (STAR 2.7.9a, Dobin et al. (2013)) was applied to align sequences to human genome *Homo sapiens* GRCh38 (annotated with GENCODE version 21). EmptyDrops (DropletUtils R package 1.16.0, Lun et al. (2019)) with FDR ≤ 0.01 was used to filter out empty droplets.

The data were further processed using R programming language (4.1.1 and 4.2.2, R Core Team (2022)) and the Seurat package (4.1.0 and 4.3.0, Hao et al. (2021)). The data were SCTransformed and integrated excluding mitochondrial and ribosomal genes prefixed by *MT‐* and *RPS‐*/*RPL‐*, if applicable (Chromium Fixed RNA Kit does not contain probes for rRNA). Further, the data went through an iterative process of quality control and filtering, including identification of doublets with DoubletFinder (2.0.3, McGinnis et al. (2019)) and removal of cells containing high levels of mitochondrial or contaminating transcripts. In the co‐culture dataset, the expression values were corrected according to the contamination estimated by SoupX package (1.5.2, Young and Behjati (2020)). Quality control process for both datasets is summarized in Figures S1B–D and S6A,B.

**FIGURE 1:**
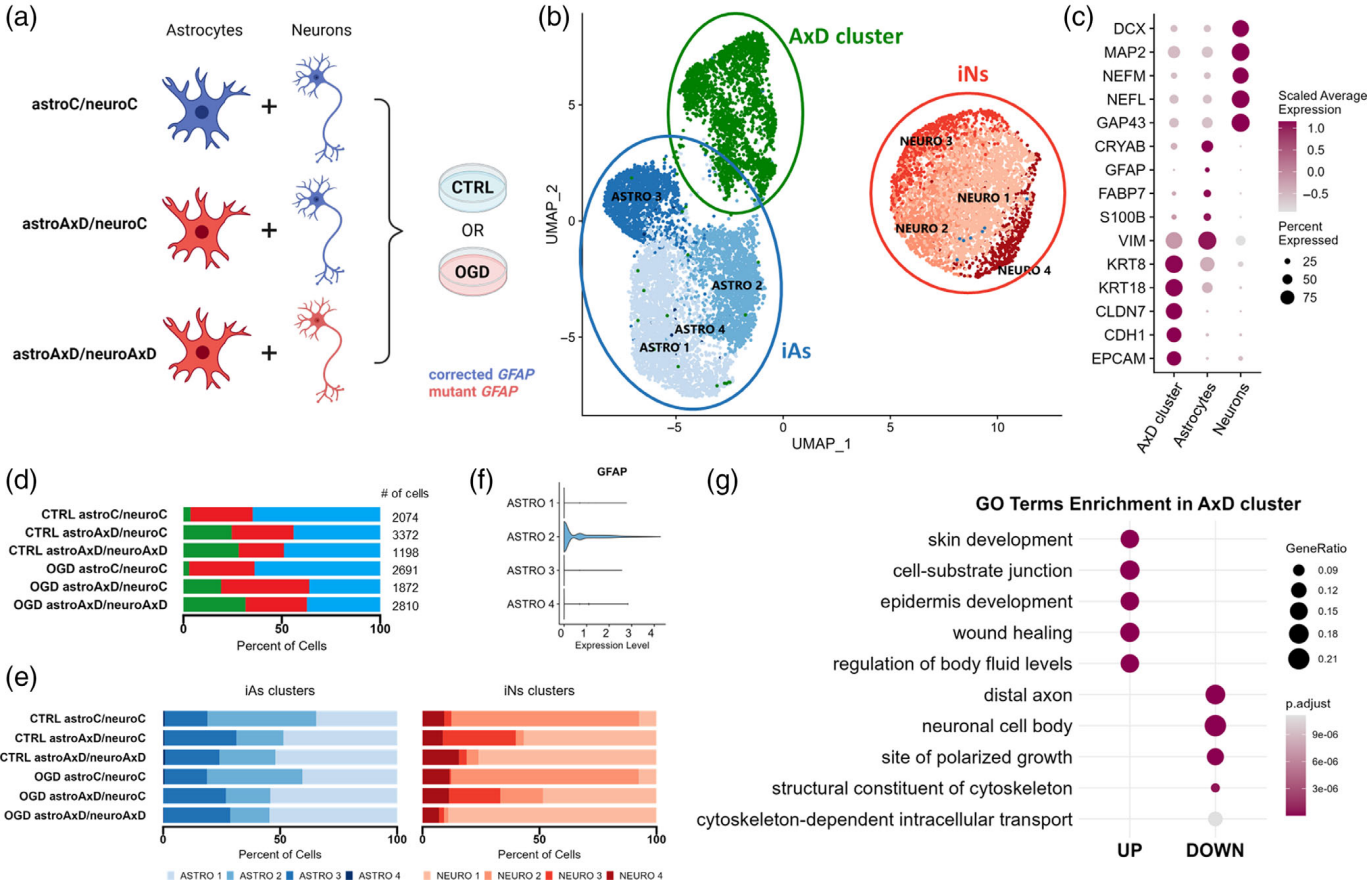
Overview of the scRNA‐seq dataset derived from astrocyte‐neuron co‐cultures. (a) Experimental design explaining co‐culture compositions and conditions entering the scRNA‐seq analysis. (b) UMAP plot showing three main cellular populations and their clusters across all six samples. AxD cluster: *n* = 2558, iAs: *n* = 6855, iNs: *n* = 4604 (c) Dotplot with selected marker genes of the cluster of the AxD cluster, iAs, and iNs. Wilcoxon test with Bonferroni correction was used to determine marker genes. (d, e) Proportional barplots showing higher abundance of the population of the AxD cluster and less mature cells in astroAxD/neuroC and astroAxD/neuroAxD samples. Oxygen–glucose deprivation (OGD) challenge did not significantly reflect in population proportions. (f) Violin plot showing that *GFAP* was expressed predominantly in the ASTRO 2 cluster. (g) Overrepresentation analysis results showing top five upregulated and downregulated GO terms distinguishing the AxD cluster from iAs and iNs (*p*_adj_ < 0.1, false discovery rate (FDR) was used to correct for multiple comparisons). astroC/neuroC, corrected co‐cultures; astroAxD/neuroC, co‐cultures with AxD astrocytes and corrected neurons; astroAxD/neuroAxD, co‐culture with AxD astrocytes and neurons; AxD, Alexander disease; CTRL, control without OGD challenge; GO, gene ontology; iAs, induced astrocytes; iNs, induced neurons; OGD, oxygen–glucose deprivation.

*NormalizeData()*, *ScaleData()*, and *SCTransform()* Seurat functions were used for normalization, scaling, and transformation. The data were visualized with UMAP and *FindNeighbors()* and *FindClusters()* functions were used to identify clusters. Cell cycle score was assigned to cells by *CellCycleScoring()* function. *FindAllMarkers()* was used to identify cluster markers using the default Wilcoxon test (Tables S4A, S7A, S8A). The three cell groups identified in co‐culture data (the AxD cell cluster, astrocytes, neurons) were clustered and analyzed separately, excluding mitochondrial and ribosomal genes. For the purposes of visualization in Figure 1, the three cell groups were merged, while maintaining clustering labels from individual analyses. Also, the merged dataset entered the analysis of cell–cell communication potential performed using CellChat (1.6.1, Jin et al. (2021)) package. Unguided and cortical organoid data were treated as separate datasets. Because of large difference between cortical control and AxD obtained with standard processing, an additional integration step was applied to overlay similar cell populations. To identify stressed cells originating in the organoid core, we applied the recently introduced Gruffi package (0.7.4, Vértesy et al. (2022)) with *neurogenesis* GO:0022008 set for negative filtering. Additionally, using Seurat *AddModuleScore*() function, we visualized expression of stress‐related gene sets from the Gene Ontology database (Gene Ontology Consortium, 2021) suggested by the authors of Gruffi—*glycolysis* GO:0006096 and *endoplasmic reticulum stress* GO:0034976 (Figure S6E–H).

The differential expression analysis (DEA) was performed on the clusters of interest using *t*‐test in *FindMarkers*(*logfc*.*threshold = 0*, *min*.*pct = 0*.*1*, *test*.*use =* “*t*”) function. The significance threshold for differentially expressed genes (DEGs) was |log_2_FC| > 0.65 and *p*_adj_ < 0.05 with Bonferroni correction. The enrichment of Gene Ontology terms (Gene Ontology Consortium, 2021) (biological process, molecular function, and cellular component) was analyzed with clusterProfiler (4.2.2 and 4.4.4, Wu et al. (2021)) package. The Gene Set Enrichment Analysis (GSEA) and Overrepresentation Analysis (ORA) were implemented with functions *gseGO*(*ont = “ALL”*, *keyType = “ALIAS”*, *minGSSize = 3*, *maxGSSize = 800*, *pvalueCutoff = 0*.*1*, *pAdjustMethod = “BH”*) and *enrichGO*(*keyType = “ALIAS”*, *OrgDb = org*.*Hs*.*eg*.*db*, *ont = “ALL”*, *pAdjustMethod = “fdr”*, *pvalueCutoff = 0*.*1*, *minGSSize = 3*). ORA of custom gene sets from Zeng et al. (2023) was performed with *enricher*() function.

Top 10 markers of each co‐culture cluster (the AxD cell cluster and iAs: log_2_FC > 1, *p*_adj_ < 0.05; iNs: log_2_FC > 0.65, *p*_adj_ < 0.05) were projected on organoid UMAPs using *Seurat::AddModuleScore*() function. Overlap of the co‐culture and organoid data was calculated using *clusterProfiler::enricher*() function. Organoid cluster markers (broad markers were considered: log_2_FC > 0.25, *p*_adj_ < 0.05) were supplied in the TERM2GENE argument, and the enrichment was calculated separately for each co‐culture cluster. The results were summarized in a matrix and a heatmap, where each row and its *p*_adj_ values correspond to one enrichment analysis. NA *p*_adj_ values were replaced by 1, and for this case the number of shared genes among cluster markers was set to 0.

### 2.21 Proteomics

Single 150‐day‐old cortical organoids were lysed in 100 mM triethylammonium bicarbonate buffer pH 8.5 and 0.2% *n*‐Dodecyl β‐D‐maltoside and resuspended thoroughly. Proteins were digested with 20 μg trypsin (Worthington) supplemented with 5 mM CaCl_2_ for 2 h at 50°C. Peptides were fractionated in 8 pH fractions using strong anion exchange (flow through, pH 11, 8, 6, 5, 4, 3, and 2) and dried in vacuo. Peptides were cleaned using in‐house manufactured C18 stagetip columns and eluates were dried in vacuo.

Samples were separated on a 20‐cm pico‐tip column (50 μm ID, New Objective) packed in‐house with C18 material (1.9 μm aquapur gold, dr. Maisch) using a two‐step 140‐min gradient that was adjusted slightly for each SAX fraction: (F1 and F2: 5%–24% ACN/0.2% FA in 80 min, and to 50% in 40 min; F3: 5%–27% ACN/0.2% FA in 80 min, and to 50% in 40 min; F4: 6%–29% ACN/0.2% FA in 80 min, and to 52% in 40 min; F5: 7%–30% ACN/0.2% FA in 80 min, and to 55% in 40 min; F6: 8%–33% ACN/0.2% FA in 80 min, and to 58% in 40 min; F7: 10%–34% ACN/0.2% FA in 80 min, and to 58% in 40 min) using an easy‐nLC 1200 system (Thermo Fisher Scientific). Peptides were electro‐sprayed directly into an Orbitrap Exploris 480 Mass Spectrometer (Thermo Fisher Scientific). The column temperature was maintained at 45°C using a column oven (Sonation). Spray voltage was set to 2.1 kV, funnel RF level at 60, and the transfer capillary temperature at 275°C. The FAIMS device was set at standard resolution and a carrier gas flow of 3.8 and alternated between CV‐45 and CV‐65. The MS was operated in DDA mode, and full scans were acquired with a resolution of 120,000 and a scan range from 450 to 1200 m/z, with an AGC target of 300% and a maximum injection time of 50 ms. Most intense precursor ions were selected for fragmentation for 2 s at a normalized collision energy (NCE) of 32%, after reaching the AGC target of 200% or maximum injection time of 200 ms. MS/MS was acquired at a resolution of 30,000, with an exclusion duration of 120 s.

RAW data files were split into two based on their FAIMS compensation voltages using FreeStyle 1.8 SP2 QF1 (Thermo Fisher Scientific) and then processed with MaxQuant (1.6.3.4, Cox and Mann (2008)), and MS2 spectra were searched with the Andromeda search engine against the SwissProt protein database of *H*. *sapiens* spiked with common contaminants. Methionine oxidation and protein *N*‐term acetylation were set as variable modifications. Trypsin was specified as enzyme and a maximum of two missed cleavages was allowed. Filtering was done at 1% false discovery rate (FDR) at the protein and peptide level. Label‐free quantification (LFQ) was performed, and “match between runs” was enabled. The data were further processed using Perseus (1.6.0.7, Tyanova et al. (2016)). Only proteins that were identified in 3 out of 4 replicates in at least one condition were considered for further analysis. Empty values were imputed from a normal distribution (width 0.3, down shift 1.8).

Differentially expressed proteins (DEPs) were identified with *t*‐test (FDR < 0.05; Table S9A). For correlation calculation, DEGs in scRNA‐seq dataset were determined in a pseudo bulk manner comparing control and AxD samples across all clusters simultaneously (Table S9B). Correlation of DEGs with DEPs was calculated using Pearson's correlation coefficient. Only genes present in the DEG as well as the DEP set, with *p*_adj_ < 0.05, were used (2521 genes).

Enrichment of biological processes from the Gene Ontology database among the upregulated and downregulated DEPs was calculated using clusterProfiler (4.4.4) R package with the function *enrichGO*(*keyType = “ALIAS”*, *OrgDb = org*.*Hs*.*eg*.*db*, *ont = “BP”*, *pAdjustMethod = “fdr”*, *pvalueCutoff = 0*.*1*, *minGSSize = 5*, *maxGSSize = 800*).

## 3 RESULTS

### 3.1 scRNA‐seq reveals a population of less differentiated cells in astrocyte‐neuron co‐cultures containing AxD astrocytes

We differentiated AxD patient‐derived iPS cells and their isogenic controls (Battaglia et al., 2019) into induced astrocytes (iAs) and induced neurons (iNs) using lentiviral transduction of Sox9 and Nfib or Ngn2, respectively (Canals et al., 2018; Zhang et al., 2013) (Figure S1A). To assess the effect of the GFAP^R239C^ mutation on the transcriptome at the single‐cell level, we applied scRNA‐seq on a co‐culture system of iAs and iNs. In individual co‐cultures, we combined iAs and iNs carrying either the corrected or the mutant *GFAP* gene, resulting in fully corrected co‐cultures (astroC/neuroC), fully mutant co‐cultures (astroAxD/neuroAxD), and a combination of mutant iAs and corrected iNs (astroAxD/neuroC) (see experimental setup in Figure 1a).

Using marker genes, the scRNA‐seq data revealed two groups of cells annotated as iNs and iAs. A third, AxD‐specific cell population (AxD cluster) appeared almost exclusively in astroAxD/neuroC and astroAxD/neuroAxD co‐cultures and was characterized by the increased expression of epithelial markers (Figure 1b–d), including *CLDN7*, *CDH1*, and *EPCAM* (Dong et al., 2018). Furthermore, Gene Ontology (GO) enrichment analysis (Figure 1g and Table S4B,C) of the AxD cluster markers showed differences in *cell‐substrate junction, structural constituent of cytoskeleton*, and *cytoskeleton‐dependent intracellular transport*. Levels of *SOX9* and *NFIB* expression suggested that the AxD cluster originated from iPS cells that had been induced into astrocytes, rather than neurons (Figure S2E). To clarify the identity of the AxD cluster, we performed an enrichment analysis using a reference study by Zeng et al. (2023), which had mapped early stages of human gastrulation by scRNA‐seq and therefore, constitutes a suitable resource of gene signatures defining cell populations during early development. This analysis revealed a substantial overlap between the AxD cluster markers and the markers of epithelial cells derived from surface ectoderm, as defined by Zeng et al. (2023) (Figure S1E). The enriched populations included diverse nonneural cell populations. The simultaneous expression of epithelial, neuronal, and astrocyte genes within the AxD cluster points to mixed identity of these cells (Figure S2C). Together, these results imply a differentiation impairment in co‐cultures containing AxD astrocytes.

Within the iAs and iNs populations, multiple clusters with varying abundance across samples were identified (Figure 1e, Figure S2, Table S4A). Four astrocyte clusters (ASTRO 1–4) included mature astrocytes (ASTRO 2) characterized by the expression of canonical astrocyte markers *GFAP* and *S100B* (Figure S2A and Figure 1f). Cells in the ASTRO 3 cluster expressed high levels of collagens, the increase of which was reported in astrocytes in vitro (Heck et al., 2003). ASTRO 1 and ASTRO 4 (Figure S2A) shared multiple markers that suggested a lower degree of maturation (*CRLF1*, *CD9*, and *ITGA7*) (Chaboub et al., 2016; Haas et al., 2017; Podergajs et al., 2016). The ASTRO 4 cluster additionally expressed cell proliferation genes (e. g., *NUSAP1* and *MKI67*). Clusters NEURO 1–3 represented different maturation states of neurons (*DCX*, *MAP2*, *NEFM*, *NEFL*; Figure 1c, Figure S2B). NEURO 1 markers included genes that are increased in neuronal precursors (*IGFBP2*, *FTL*, *SERF2*, and *VIM*) (Freed et al., 2008; Kirkcaldie & Dwyer, 2017; Shen et al., 2019). NEURO 3 shared some of these markers and expressed also genes involved in hormone secretion (*PTH2*, *UCN*, and *TRH*). Interestingly, while NEURO 1 was more abundant in astroAxD/neuroAxD co‐cultures (Figure 1e), NEURO 3 was enriched in astroAxD/neuroC co‐cultures, suggesting unique gene expression signatures of corrected and AxD neurons in co‐cultures with AxD astrocytes. The NEURO 2 cluster, enriched in astroC/neuroC co‐cultures (Figure 1e, Figure S2B), was characterized by expression of *NNAT*, encoding for neuronatin, a proteolipid membrane protein expressed in the developing brain (Pitale et al., 2017), as well as *SCG2*, *PCSK1*, and *SYT4* genes involved in neuropeptide processing and secretion (Fischer‐Colbrie et al., 1995; Wang et al., 2017; Zhang et al., 2009), indicating their more differentiated state compared to NEURO 1 and NEURO 3. The NEURO 4 cluster was annotated as peripheral neurons (*PHOX2B* and *ISL1*) (Lin et al., 2021) and was represented similarly across conditions. Overall, the analysis of cell populations in the scRNA‐seq co‐culture data showed the presence of a specific AxD cluster and an increased proportion of less mature astrocytes and neurons in co‐cultures that contained AxD astrocytes.

### 3.2 Impaired astrocyte differentiation in co‐cultures containing AxD astrocytes

**FIGURE 2:**
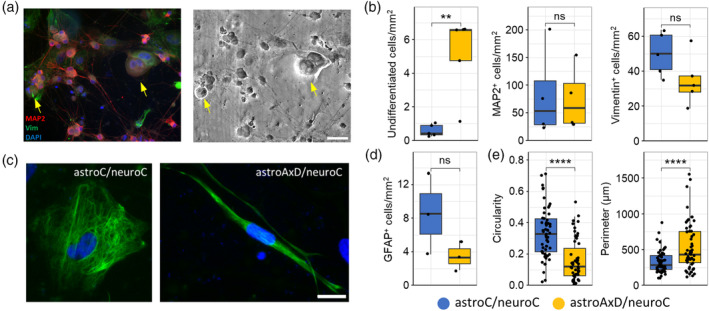
Immunocytochemistry identified less mature cells in astroAxD/neuroC co‐cultures. (a) Co‐cultures of iAs and iNs were labeled with antibodies against MAP2 (red), vimentin (green), and nuclei were visualized with DAPI (blue). Arrows point to undifferentiated cells; scale bar, 50 μm. (b) Number of undifferentiated cells, MAP2^+^, and vimentin^+^ cells. *n* = 5, 4, and 5 independent sets of astroC/neuroC and astroAxD/neuroC cultures. Wilcoxon test (undifferentiated cells) and *t*‐test (MAP2, vimentin) were used for statistical comparison. (c) Representative image of GFAP^+^ astrocytes (GFAP, green; DAPI, blue); scale bar, 20 μm. (d) Number of GFAP^+^ astrocytes, *n* = 3 independent sets of astroC/neuroC and astroAxD/neuroC cultures. *T*‐test was used for statistical comparison. (e) Circularity and perimeter of GFAP^+^ signal for individual astrocytes (astroC/neuroC: *n* = 55, astroAxD/neuroC: *n* = 55). Wilcoxon test was used for statistical comparison. ns: not significant, **p ≤ 0.01; ****p ≤ 0.0001. In b, d, and e Shapiro–Wilk test was used for normality assessment. astroC/neuroC, corrected co‐cultures; astroAxD/neuroC, co‐cultures with AxD astrocytes and corrected neurons; iAs, induced astrocytes; iNs, induced neurons.

Since the single‐cell transcriptomics data showed the presence of less mature astrocytes and neurons in astroAxD/neuroC co‐cultures, we further characterized the cell populations by immunocytochemistry using antibodies against MAP2 to visualize neurons, and antibodies against vimentin to visualize astrocytes (Figure 2a). The astroC/neuroC and astroAxD/neuroC co‐cultures contained comparable numbers of vimentin‐positive astrocytes and MAP2‐positive neurons (Figure 2b). The density of undifferentiated cells (identified by their spherical morphology, the lack of cellular processes and distinctly larger size than neurons) was higher in co‐cultures containing AxD astrocytes (Figure 2a,b). The proportion of cells identified across both genotypes using immunofluorescence was 9/7/0.5 (neurons/astrocytes/undifferentiated cells), while the neuron/astrocyte/AxD cluster ratio was identified as 3/5/1 by scRNA‐seq (Figure 1b,d). This implies an increased loss of neurons during the preparation of single‐cell suspensions, as expected due to their more fragile nature (Cuevas‐Diaz Duran et al., 2022; Lafzi et al., 2018), resulting in an overrepresentation of undifferentiated cells. GFAP immunolabeling and cell quantification showed no statistically significant difference in the number of GFAP‐positive cells between astroC/neuroC and astroAxD/neuroC (Figure 2c,d). As GFAP is expressed also in RG, we next quantitatively assessed selected morphological features of the GFAP‐positive cells. We found that GFAP‐positive cells in co‐cultures containing AxD astrocytes exhibited lower circularity and a larger perimeter (Figure 2e), indicative of a more RG‐like morphology, that is, a less differentiated astrocyte phenotype (Liour & Yu, 2003). Jointly, the immunocytochemical analysis of the co‐cultures indicated a lower degree of differentiation of co‐cultures containing AxD astrocytes.

### 3.3 The AxD mutation affects the development and interactions of iAs and iNs

**FIGURE 3:**
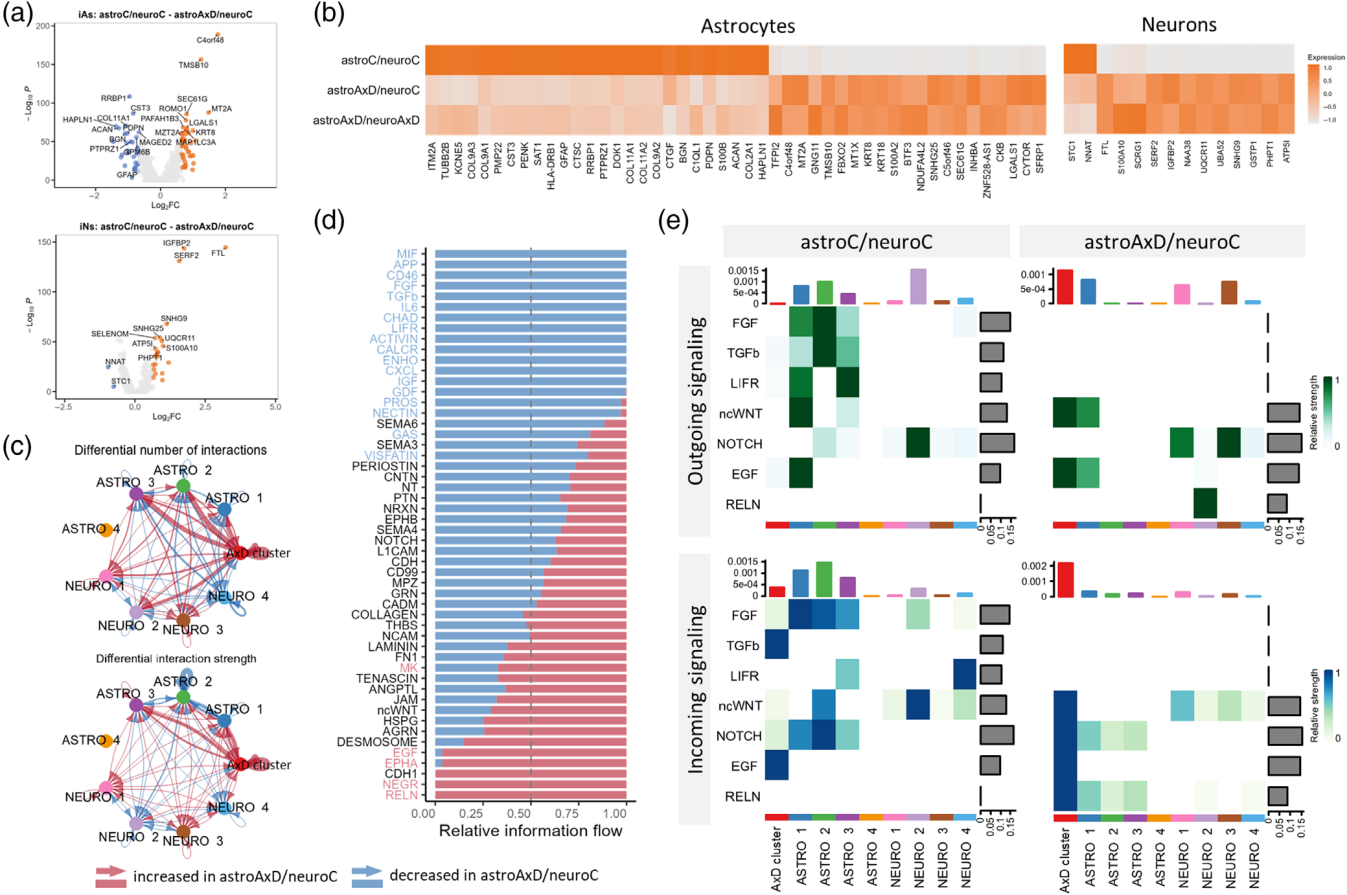
Results of differential expression analysis of iAs and iNs and cell–cell interaction analysis. (a) Volcano plot showing DEGs between astroC/neuroC and astroAxD/neuroC astrocytes and neurons (|log_2_FC| > 0.65 and *p*_adj_ < 0.05, *t*‐test was used to calculate DEGs, Bonferroni correction was used to correct for multiple comparisons). (b) Heatmap shows expression of DEGs shared between comparisons of astroC/neuroC with astroAxD/neuroC and with astroAxD/neuroAxD. (c) Differential number of interactions (top) and differential interaction strength (bottom) between astroC/neuroC and astroAxD/neuroC defined by CellChat analysis (red—increase in astroAxD/neuroC, blue—decrease in astroAxD/neuroC). (d) Information flow chart from the CellChat analysis of cell–cell interactions presents the pathways identified in both conditions. Significantly dysregulated pathways (paired Wilcoxon test, *p*‐value < 0.05) are highlighted in colors (red—increase in astroAxD/neuroC, blue—decrease in astroAxD/neuroC). (e) Heatmap divided to outgoing (ligands) and incoming (receptors) signaling patterns shows selected neurodevelopmental and astrogenesis‐related pathways that were changed in astroAxD/neuroC compared to astroC/neuroC co‐cultures. astroC/neuroC, corrected co‐cultures; astroAxD/neuroC, co‐cultures with AxD astrocytes and corrected neurons; astroAxD/neuroAxD, co‐culture with AxD astrocytes and neurons; AxD, Alexander disease; DEGs, differentially expressed genes; iAs, induced astrocytes; iNs, induced neurons.

To identify the effect of the GFAP^R239C^ mutation on gene expression in individual cell types in the co‐culture systems, we performed DEA. First, we took advantage of the astroAxD/neuroC co‐cultures, where only astrocytes carry the *GFAP* mutation. The mutant *GFAP* astrocytes downregulated genes such as *GFAP*, *S100B*, *ACAN*, and *PTPRZ1*, indicating impaired astrocyte differentiation. The upregulation of metallothioneins *MT2A* and *MT1X* suggested an increased stress response (Juárez‐Rebollar et al., 2017; Ruttkay‐Nedecky et al., 2013) in astroAxD/neuroC co‐cultures (Figure 3a,b). These genes were differentially expressed in AxD astrocytes regardless of the genotype of the co‐cultured neurons (Figure 3b, Table S5A). In total, we identified 21 upregulated and 26 downregulated genes shared between the two comparisons (i.e., astroAxD/neuroC vs. astroC/neuroC and astroAxD/neuroAxD vs. astroC/neuroC, Figure S4A). Importantly, assessing the gene expression changes in neurons, we found that regardless of the neuronal genotype, the AxD astrocytes affected expression of neuronal genes (Figure 3a,b, Figure S4A, Table S5C). The downregulated genes included *NNAT* involved in development and calcium signaling (Pitale et al., 2017) and glutathione S‐transferase *GSTP1*. The genes upregulated in AxD astrocyte‐containing co‐cultures included genes participating in oxidative stress response (*STC1*, Bonfante et al. (2020)), ion channel function (*S100A10*, Seo and Svenningsson (2020)), protein aggregation (*SERF2*, Stroo et al. (2023)), ubiquitination (*UBA52*, Kobayashi et al. (2016)), and mitochondrial function (*ATP5I*, *UQCR11*).

To assess whether the genotype of neurons affected the mutant *GFAP* astrocytes, we analyzed differential gene expression between astrocytes from astroAxD/neuroC and astroAxD/neuroAxD co‐cultures (Figure S4B, Table S5A). This comparison revealed several genes downregulated in astroAxD/neuroAxD co‐cultures, including VGF nerve growth factor inducible (*VGF*) and synaptotagmin 4 (*SYT4*). These differences were reflected in GO enrichment analysis as synapse and cell–cell signaling (Figure S4C, Table S5B). Addressing the difference between AxD and corrected isogenic control neurons co‐cultured with AxD astrocytes, we found upregulation of neurodevelopmental genes (*SOX4* and *PCDH17*) (Braccioli et al., 2018; Peek et al., 2017) and the mature neuronal marker gene (*NEFM*) in astroAxD/neuroAxD. AxD neurons showed also downregulation of genes coding for neuropeptides (*CRH*, *TAC1*, *PTH2*, and *UCN*), synaptic proteins (*SYT4*), and genes involved in hormone secretion (*TRH*, *SCG5*) (Figure S4B, Table S5C). Thus, both comparisons suggested altered cell–cell communication between astrocytes and neurons in astroAxD/neuroAxD co‐cultures, possibly due to immaturity of AxD neurons.

Given the indications of cell–cell interaction changes in AxD co‐cultures, we further focused on the astroAxD/neuroC co‐cultures and investigated the cell–cell interaction potential of the AxD cluster, iAs, and iNs using the CellChat analysis (Jin et al., 2021). We identified increased number and strength of interactions in astroAxD/neuroC co‐culture compared to astroC/neuroC (Figure 3c), involving specifically the AxD cluster. As the CellChat analysis controlled for different size of cell clusters, this suggested an increased signaling activity of the AxD cluster. The ASTRO 2 cluster representing mature astrocytes was less involved in cell communication in astroAxD/neuroC. The ASTRO 4 cluster contained very few cells for any ligands and receptors to be reliably detected. Multiple pathways were dysregulated in astroAxD/neuroC compared to astroC/neuroC (Figure 3d). For instance, astroAxD/neuroC upregulated RELN and EGF pathways. *RELN* expression was activated in the NEURO 2 cluster, and astrocytes and the AxD cluster expressed *RELN* receptors *ITGA3* and *ITGB1* (Figure 3e, Figure S3B). The interactions between reelin and integrins participate in neuronal migration along RG during corticogenesis (Belvindrah et al., 2007; Dulabon et al., 2000). Thus, these results indicate that the isogenic control neurons signal to AxD astrocytes, targeting mainly their less differentiated, more RG‐like states, while such interactions with less differentiated astrocytes were absent in the astroC/neuroC and astroAxD/neuroAxD co‐cultures (Figure 3e, Figure S3C,D). EGF signaling (HBEGF/AREG‐(EGFR+ERBB2)), which has a prominent role in gliogenesis (Zhang et al., 2023), was enhanced in the astroAxD/neuroC AxD cluster (Figure 3e, Figure S3A,B). EGFR^+^ progenitor cells are present in the developing cortex around the period of the gliogenic switch (Fu et al., 2021), and therefore, the increased EGF signaling potential in the astroAxD/neuroC AxD cluster would be compatible with delayed or halted differentiation of the AxD cluster. Other pathways associated with astrogenesis (Voss et al., 2023; Zarei‐Kheirabadi et al., 2020) and mediating communication between astrocytes and neurons in astroC/neuroC were absent in astroAxD/neuroC (Figure 3e). These included FGF (pair FGF2‐FGFR1), TGFb (ligands TGFB1 and TGFB2), and LIFR (LIF‐(LIFR+IL6ST), CLCF1‐(CNTFR+LIFR)) (Figure 3e, Figure S3A). Additionally, in astroC/neuroC, we detected the ASTRO 1 immature astrocytes, more mature ASTRO 2 astrocytes, and NEURO 2 as the main senders and receivers of the non‐canonical WNT (ncWNT) signaling (mainly WNT5B‐FZD3/FZD6). In astroAxD/neuroC this pathway was enhanced in the AxD cluster. Similarly, the cells of the AxD cluster were also the main recipients of NOTCH signaling coming from neuronal clusters (mainly DLL3‐NOTCH2) (Figure 3e, Figure S3A,B). Both the ncWNT and Notch signaling play an important role in neurodevelopment, with WNT5B participating in cytoskeleton rearrangement, mechanosensing, and neural tissue patterning (Suthon et al., 2021) and Notch maintaining the pool of progenitors and being dependent of astrocyte intermediate filaments (Lampada & Taylor, 2023; Suthon et al., 2021; Wilhelmsson et al., 2012).

Together, these results support the concept of impaired astrocyte differentiation and point to an increased stress level in mutant *GFAP* co‐cultures. Moreover, neuronal development was found to be affected by iAs with the AxD mutation, and the cell–cell interaction analysis showed the absence of or impaired signaling potential related to astrogenesis and neurodevelopment in astroAxD/neuroC astrocyte‐neuron co‐cultures.

### 3.4 OGD challenge enhances the effect of the AxD mutation

**FIGURE 4:**
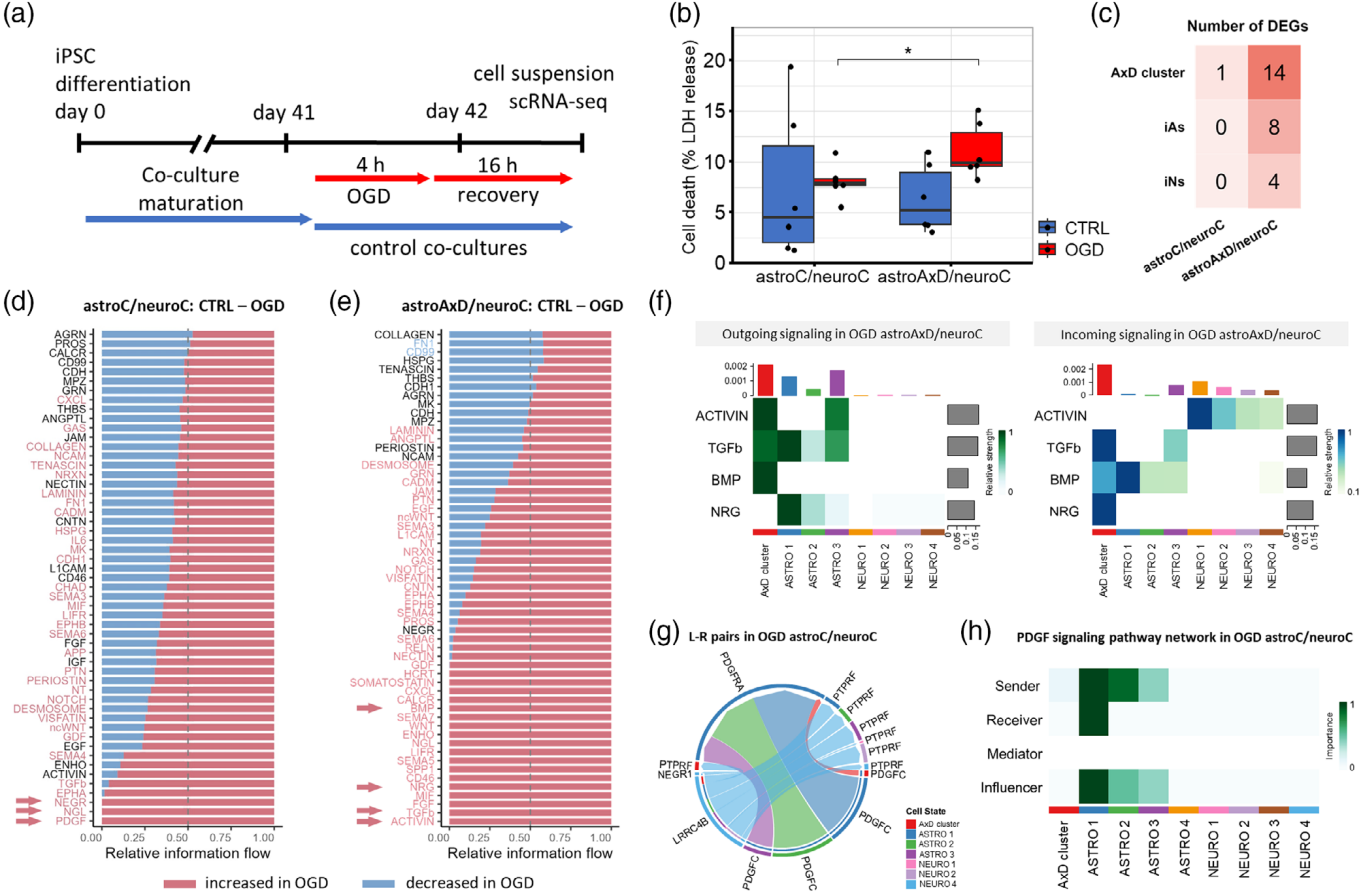
The OGD challenge enhanced the effect of the GFAP mutation in the AxD cluster. (a) A scheme of the timeline of the OGD challenge. (b) LDH assay measuring cell death showed increased LDH release in OGD astroAxD/neuroC co‐culture compared to OGD astroC/neuroC and astroAxD/neuroC without stress, *n* = 6 sets of cultures of CTRL and OGD, astroC/neuroC and astroAxD/neuroC. *T*‐test was used for statistical comparison, Shapiro–Wilk test was used for normality assessment, *p < 0.05. (c) Heatmap summarizing numbers of DEGs showing that the AxD cluster was the most affected by the OGD challenge (|log_2_FC| > 0.65 and *p*_adj_ < 0.05; *t*‐test with Bonferroni correction). (d, e) Information flow chart showing pathways affected by the OGD challenge in astroC/neuroC (d) and astroAxD/neuroC (e) co‐cultures. Significant change is marked by colors: red—increased in OGD, blue—decreased in OGD; paired Wilcoxon test, *p*‐value < 0.05. Arrows highlight pathways included in panels f–h. (f) Heatmap of selected pathways that are enriched in astroAxD/neuroC co‐cultures after OGD challenge. (g) Ligand‐receptor pairs of three pathways uniquely enriched in astroC/neuroC co‐cultures after OGD. Clusters of senders and receivers are distinguished by colors. (h) Detailed heatmap of PDGF signaling pathway shows roles of individual clusters in this signaling based on statistical and network analysis by CellChat. astroC/neuroC, corrected co‐cultures; astroAxD/neuroC, co‐cultures with AxD astrocytes and corrected neurons; AxD, Alexander disease, CTRL, control co‐culture without stress; DEGs, differentially expressed genes; iAs, induced astrocytes, iNs, induced neurons; LDH, lactate dehydrogenase; L‐R pairs, ligand‐receptor pairs; OGD, oxygen–glucose deprivation.

To investigate the susceptibility and adaptation of the co‐cultures to stress, we subjected them to a mild OGD challenge for 4 h followed by a 16 h long recovery period (Figure 4a). Cell death measured 2 h after the challenge as a release of lactate dehydrogenase (LDH) indicated no OGD‐induced cell death in astroC/neuroC co‐cultures, while in astroAxD/neuroC co‐cultures an increase in OGD‐induced cell death was found (Figure 4b).

We collected and analyzed samples after the recovery period and performed scRNA‐seq. The OGD challenge was not significantly reflected in population proportions (Figure 1d,e). To address the effect of OGD on individual cell types, we performed DEA comparing the control (unchallenged) and the respective OGD samples in astroC/neuroC and astroAxD/neuroC co‐cultures. In corrected iAs and iNs, no genes were differentially expressed (Figure 4c), and only a single differentially expressed gene was detected in the corrected cells within the AxD cluster. In contrast, several genes were downregulated in OGD in all three cell populations in the astroAxD/neuroC co‐cultures (Figure 4c, Figure S4D, Table S6A). The most affected cell type appeared to be the cells of the AxD cluster. In these cells 14 genes were downregulated after the OGD challenge. Using gene set enrichment analysis (GSEA) we identified an upregulation of the GO terms *cell adhesion molecule binding*, *anchoring junction*, and *plasma membrane region* (Figure S4E, Table S6B), and a downregulation of GO terms related to mitochondrial function and respiration (e.g., *mitochondrial protein‐containing complex*, *cellular respiration*), which might reflect the increased sensitivity of cells of the AxD cluster to the OGD challenge.

The analysis of cell–cell interactions with CellChat showed that 18 signaling pathways were exclusively upregulated in astroAxD/neuroC co‐cultures after OGD and recovery, compared to the astroAxD/neuroC co‐cultures without OGD (Figure 4d,e), many with cells of the AxD cluster and immature astrocytes as signal sending or signal receiving cells. These included TGFβ‐related pathways (ACTIVIN, TGFb, and BMP), along with neuregulin (NRG) signaling previously linked to the differentiation of RG into astrocytes (Schmid et al., 2003) (Figure 4f). In astroC/neuroC co‐cultures, only three pathways were exclusively detected after OGD and recovery, including PDGF signaling, specifically PDGF‐C astrocyte‐astrocyte signaling via PDGFRα (Figure 4g,h). Interestingly, the PDGF‐C signaling to PDGFRα, which was previously associated with the astrocyte response to stress (Miyata et al., 2014), was absent in astroAxD/neuroC co‐cultures, indicating reduced adaptation of AxD astrocytes to OGD‐induced stress.

### 3.5 scRNA‐seq reveals altered differentiation in AxD unguided neural organoids

**FIGURE 5:**
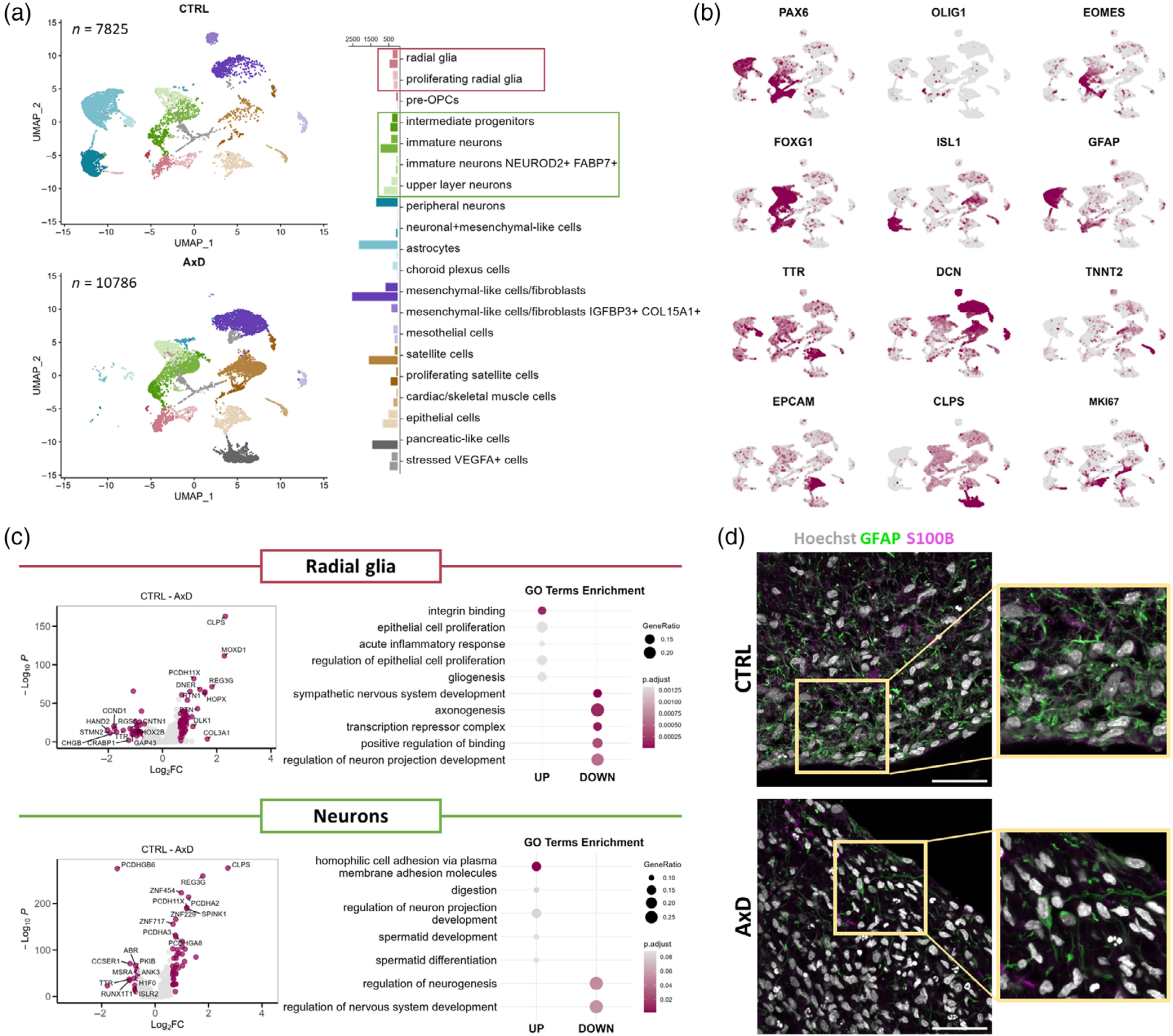
GFAP mutant unguided neural organoids partially diverged their differentiation into other than neuroectodermal lineage and did not develop astrocyte‐like cells. (a) UMAP plot showing various cell types that were identified in 165 days old unguided organoids, split to conditions. Legend includes a barplot depicting abundance (absolute cell counts) of clusters in each condition (top bar = CTRL, bottom bar = AxD). (b) Selected markers highlighting clusters of neuroectodermal lineage, as well as off‐target populations that were overrepresented in AxD organoids. (c) DEA was performed on clusters of radial glia (in a highlighted with a magenta rectangle) and neuronal clusters (in a highlighted with a green rectangle) comparing CTRL and AxD. Volcano plots show DEGs (|log_2_FC| > 0.65 and *p*_adj_ < 0.05; *t*‐test with Bonferroni correction). GO overrepresentation analysis was performed on the DEGs, with FDR used to correct for multiple comparisons and *p*_adj_ < 0.1 used as significance threshold for the results. (d) Immunofluorescent microscopy images showing a Hoechst (nuclei), GFAP, and S100B staining of Day 165 CTRL and AxD unguided organoids. Scale bar, 50 μm. AxD, Alexander disease; CTRL, control; DEA, differential expression analysis; DEGs, differentially expressed genes; FDR, false discovery rate; GO, gene ontology; log_2_FC, log_2_ fold change; *p*_adj_, adjusted *p*‐value; pre‐OPCs, pre‐oligodendrocyte progenitor cells.

The differentiation defect observed in GFAP‐mutant astrocyte‐neuron co‐cultures prompted us to investigate the effect of the GFAP^R239C^ mutation in an organoid model, which better mimics the in vivo development. To do so, we generated unguided neural organoids (Lancaster et al., 2013; Ormel et al., 2018) from the same AxD iPS cells and their isogenic corrected controls as were used for the astrocyte‐neuron co‐cultures. We cultured the organoids until Day 165, by which time several cell populations, including astrocytes and neurons, had emerged, and subjected them to scRNA‐seq (Figure S5A). Using marker genes, we annotated 20 cell populations (Figure 5a,b, Figure S6C, Table S7A), including cell types of the neural lineage, such as RG (*PAX6*, *SOX2*), pre‐oligodendrocyte progenitor cells (pre‐OPCs; *OLIG1*, *EGFR*, *DLL3*) (Van Bruggen et al., 2022), intermediate progenitors (*EOMES*) (Kanton et al., 2019), and CNS excitatory neurons (*NEUROD2*, *NEUROD6*, and *GRIA2*) (Kanton et al., 2019). We also identified a peripheral neuron‐like population (*PHOX2B*, *PRPH*, and *ISL1*) (Lin et al., 2021), a population of choroid plexus cells (*TTR*, *TRPM3*, and *CA2*) (Pellegrini et al., 2020), and a population of astrocytes expressing *GFAP*, *S100B*, and *FOXJ1* (Jacquet et al., 2009; Li, Floriddia, et al., 2018). We detected cells of mesodermal origin, that is, mesenchymal‐like cells/fibroblasts (*DCN* and *COL1A1*) (Pfau et al., 2024), satellite cells (*MYF5*and *PAX7*) (Motohashi & Asakura, 2014), muscle cells (*MYOG* and *TNNT2*) (Liu et al., 2012), and mesothelial cells (*LRRN4* and *UPK3B*) (Kanamori‐Katayama et al., 2011). Additionally, there were populations of epithelial cells (*EPCAM* and *ELF3*) (Dong et al., 2018), pancreatic‐like acinar cells (*CLPS*, *CTRB1*, *CTRB2* and *CPA2*) (Ma et al., 2023), and a cluster of cells characterized by increased glycolysis and endoplasmic reticulum stress (*VEGFA*, *DDIT4*; Figure S6E,F), known to originate in the organoid center where there is a limited supply of nutrients and oxygen (Vértesy et al., 2022). RT‐qPCR of control and AxD unguided neural organoids revealed reduced expression of markers of choroid plexus cells (*TRPM3*), and neurons (*PHOX2B*), and increased expression of markers of muscle cells (*MYF5*), and pancreatic acinar cells (*CLPS*) in the AxD organoids, reflecting changes in the abundance of the cell populations as detected by scRNA‐seq (Figure S7A).

Importantly, the AxD unguided neural organoids showed only very small number of peripheral neurons, astrocytes, pre‐OPCs, and choroid plexus cells, and they were enriched for the acinar cells and the mesenchymal‐like populations (Figure 5a), suggesting an altered differentiation trajectory in AxD organoids. To explore the potential cause of the differentiation defect, we focused on the progenitors giving rise to neurons as well as astrocytes, and we performed DEA on the clusters of RG comparing AxD and control unguided organoids (Figure 5c, Table S7B). The DEGs downregulated in AxD RG included neuronal markers *GAP43* and *STMN2*, which were also reflected in GO enrichment analysis as *axonogenesis* and *regulation of neuron projection development* (Figure 5c, Table S7C,D). Interestingly, outer RG markers *MOXD1* and *HOPX* were upregulated in AxD RG, along with pancreatic genes *CLPS* and *REG3G*. *Integrin binding*, *epithelial cell proliferation*, and *gliogenesis* belonged to the GO terms upregulated in AxD RG. In addition, DEA of the neuronal clusters showed dysregulation of several protocadherin genes (e.g., *PCKDHA2*, *PCDHGA8*, and *PCDHGB6*) that play a role in neurodevelopment (Peek et al., 2017), as well as pancreatic genes *CLPS* and *REG3G* (Figure 5c, Table S7E). The GO enrichment analysis of neuronal DEGs showed upregulation of homophilic cell adhesion via plasma membrane adhesion molecules and downregulation of regulation of neurogenesis in AxD unguided organoids (Table S7F,G).

Immunofluorescence analysis showed the presence of GFAP^+^ cells in both control and AxD unguided neural organoids (Figure 5d). To assess the abundance of astroglial cells between control and AxD organoids, we performed immunocytochemistry for both GFAP and S100B, another marker of astrocytes (Haan et al., 1982). The AxD organoids showed a reduction in the GFAP immunoreactivity (Figure 5d), a finding supporting the transcriptomics data that showed a reduced number of astrocytes in AxD organoids. Together, these results suggest that in the AxD unguided organoids, mesodermal and endodermal differentiation was favored, while the neuroectoderm‐derived cells failed to achieve the same degree of differentiation as they did in control organoids.

### 3.6 scRNA‐seq and proteomics analyses reveal altered differentiation of AxD cortical organoids

**FIGURE 6:**
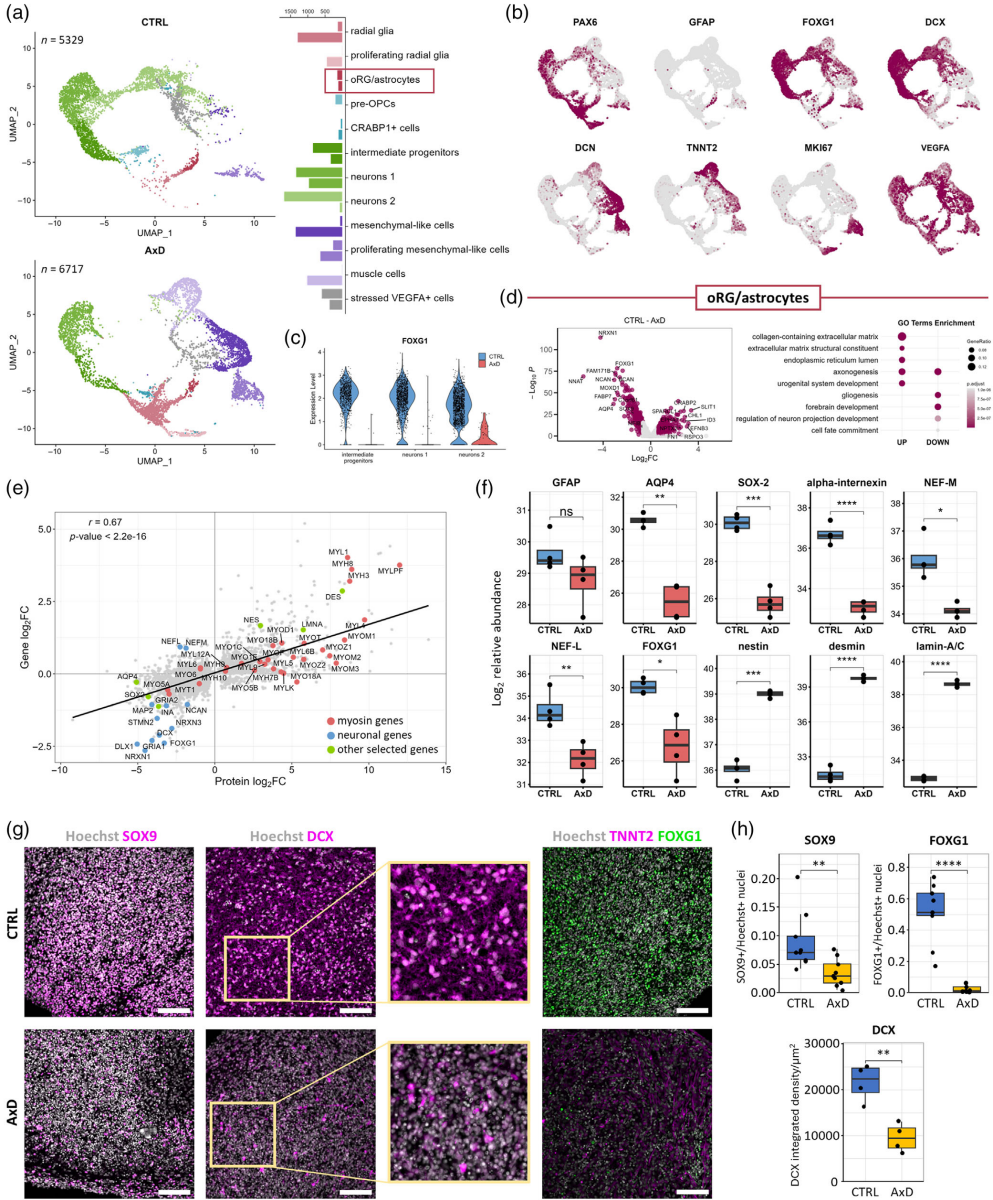
GFAP mutant cortical organoids showed delayed development and were enriched for mesoderm‐derived cell populations. (a) UMAP plot showing various cell types that were identified in 165 days old cortical organoids, split to conditions. Legend includes barplot depicting abundance (absolute cell counts) of clusters in each condition (top bar = CTRL, bottom bar = AxD). (b) Selected markers highlighting clusters of neuroectodermal lineage, as well as off‐target populations that were overrepresented in AxD organoids. (c) Expression plot of telencephalic marker *FOXG1* showing only limited expression in neuronal clusters in the AxD organoids. (d) DEA was performed on the cluster of oRG/astrocytes (in a highlighted with magenta rectangle) comparing CTRL and AxD. Volcano plot shows DEGs (|log_2_FC| > 0.65 and *p*_adj_ < 0.05; *t*‐test with Bonferroni correction). GO overrepresentation analysis was performed on DEGs with FDR used to correct for multiple comparisons and *p*_adj_ < 0.1 used as significance threshold for the results. Top five upregulated and downregulated terms are shown in the dotplot. (e) DEGs (*p*_adj_ < 0.05) and DEPs (FDR < 0.05) were plotted against each other, with fitted line, Pearson's correlation coefficient (r) and statistical significance. Myosin genes are highlighted in red, selected neuronal genes are highlighted in blue, and other genes of interest are highlighted in green. (f) Normalized intensities of selected proteins were compared in CTRL and AxD organoids using *t*‐test; ns: not significant, *p ≤ 0.05; **p ≤ 0.01; ***p ≤ 0.001; ****p ≤ 0.0001. (g) Immunofluorescent microscopy images showing SOX9, DCX, FOXG1, and TNNT2 for Day 165 CTRL and AxD cortical organoids. Nuclei are stained by Hoechst; scale bar, 100 μm. (h) Quantification of immunofluorescent signal of SOX9 and FOXG1 proteins in ratio to nuclei staining with Hoechst. Data points represent three images taken from three individual organoids. DCX integrated density was measured from four organoids per genotype and presented as integrated density per μm^2^. No difference in the Hoechst signal was observed. Shapiro–Wilk test was used to determine normal distribution of the data Wilcoxon test (SOX9, FOXG1) and *t*‐test (DCX) were used to compare CTRL and AxD samples. AxD, Alexander disease; CTRL, control; DEA, differential expression analysis; DEGs, differentially expressed genes; DEPs, differentially expressed proteins; FDR, false discovery rate; GO, gene ontology; log_2_FC, log_2_ fold change; oRG, outer radial glia; *p*_adj_, adjusted *p*‐value; pre‐OPCs, pre‐oligodendrocyte progenitor cells.

Since unguided neural organoids are characterized by their relatively wide differentiation capabilities, we aimed to investigate whether the neural lineage commitment defect in AxD organoids could be rescued by dual SMAD inhibition (inhibition of BMP and TGFβ pathways), a method known to induce neuroectoderm and generate cortical organoids (Yoon et al., 2019). We generated AxD and isogenic control cortical organoids and performed scRNA‐seq on 165 days old organoids (Figure S5A). This revealed, as expected (Yoon et al., 2019), a lower diversity of cell populations compared to unguided neural organoids (Figure 6a,b, Figure S6D,G,H, Table S8A). We identified cell populations representing outer radial glia (oRG)/astrocytes (*TNC*, *HOPX*, *FABP7*, *SOX9*, and *AQP4*), and neurons at different stages of maturation (*DCX*, *STMN2*, and *GRIA2*). AxD neuronal populations were strongly reduced and the neurons that did develop lacked expression of the forebrain marker *FOXG1* (Figure 6c). The AxD cortical organoids lacked pre‐OPCs (*GSX2*, *ASCL1*, *EGFR*, *SMOC1*, and *HES6*) (Van Bruggen et al., 2022), were strongly enriched for RG (*SOX2* and *PAX6*), and, in contrast to control organoids, contained proliferating RG (*TOP2A* and *MKI67*). We observed nonneural differentiation in AxD cortical organoids. For example, compared to controls, the AxD cortical organoids contained much larger populations of mesenchymal‐like (*DCN*) and muscle cells (*TNNT2*; Figure 6a), indicating a differentiation defect as observed in the AxD unguided neural organoids.

To address how the development of astrocytes was affected in AxD cortical organoids, we performed DEA on the AxD and control oRG/astrocyte populations (Figure 6d, Table S8B). The most prominently downregulated genes in AxD included synaptic protein *NRXN1*, neurogenesis marker *NNAT*, forebrain transcription factor *FOXG1*, and astrocyte markers *NCAN*, *BCAN*, *FABP7*, and *AQP4*. Importantly, gliogenic transcription factors *SOX9* and *NFIB* were also downregulated in AxD oRG/astrocyte population. Among the upregulated genes in AxD cortical organoids were *CHL1*, encoding a neural cell adhesion protein, extracellular matrix components *FN1* and *SPARCL1*, and retinol binding *CRABP2*. The upregulated genes in AxD cortical organoids were reflected in GO enrichment analysis as *collagen‐containing extracellular matrix*, *endoplasmic reticulum lumen*, and *axonogenesis*, whereas the downregulated GO terms included *gliogenesis*, *forebrain development*, and *cell fate commitment* (Figure 6d, Table S8C,D). We validated these findings with RT‐qPCR, detecting a decrease in the expression of neurodevelopmental genes (*NCAN* and *NNAT*) and gliogenesis‐related genes (*AQP4* and *NFIA*), and an increase in the expression of muscle cell marker *TNNT2* (Wei & Jin, 2016) (Figure S7B). The CellChat analysis performed on cell populations of neural lineage in the cortical organoids showed—similar to co‐cultures of AxD astrocytes and isogenic corrected neurons after OGD challenge—dysregulation of TGFβ signaling (represented by ACTIVIN, TGFb) in AxD organoids (Figure S7C,D). PDGF signaling (PDGFD and PDGFA via PDGFRB) was downregulated in AxD organoids and did not involve the oRG/astrocyte cluster as signal senders. Other dysregulated pathways included EGF, NOTCH (ligands DLL1/3 and JAG1 and receptors NOTCH1‐3), FGF, RELN, HH (Hedgehog), and the ncWNT pathways, pointing to a developmental impairment in AxD organoids.

To determine differences in protein abundance between control and AxD cortical organoids, we performed mass spectrometry on 150‐day‐old cortical organoids. Comparison of transcriptomic and proteomic data showed a significant positive correlation between the DEGs and DEPs (Pearson's correlation coefficient = 0.67, *p*‐value < 2.2 × 10^−16^; Figure 6e, Table S9A,B). Among the upregulated proteins in the AxD cortical organoids, we identified several myosins and other muscle‐related proteins, such as desmin (Figure 6e,f, Figure S7E). Lamin A/C was also upregulated in AxD organoids. The upregulation of lamin A/C was reported in both rat and *Drosophila* models of AxD as well as in human AxD tissue (Hagemann et al., 2021; Wang et al., 2018), and it can also reflect higher abundance of muscle cells in AxD organoids (Röber et al., 1989). In line with the observation of impaired astrogenesis and neurogenesis, we identified GFAP, AQP4, SOX2, and the neuronal proteins α‐internexin, neurofilament light chain (NF‐L) and neurofilament medium chain (NF‐M) as less abundant in AxD cortical organoids (Figure 6f). The DEPs reflected in GO enrichment analysis as synapse and neuronal function‐related terms (e.g., *neuron development*, *synaptic signaling*) were downregulated in AxD cortical organoids, while muscle development‐related terms were upregulated (Figure S7F). Overall, the proteomics data revealed a reduced abundance of neural proteins and an increase of muscle proteins, thereby confirming the transcriptomics data and supporting the concept of a reduced neural differentiation and off‐target differentiation into muscle cells.

To compare the number of astroglial cells between control and AxD cortical organoids, we performed immunocytochemistry using antibodies against SOX9, a marker of astrocytes and RG (Sun et al., 2017). The AxD cortical organoids showed a reduction in the SOX9^+^ immunoreactivity (Figure 6g,h), which is consistent with the transcriptomics results showing a prominent reduction in the number of astrocytes in AxD cortical organoids. Immunocytochemical analysis with antibodies against DCX, an early neuronal marker (Sarnat, 2015), showed decreased DCX immunoreactivity in AxD compared to control organoids (Figure 6g,h). This implicates that astrogenesis and neurogenesis are both decreased in AxD organoids. In comparison to control organoids, AxD organoids exhibited fewer cells positive for the forebrain marker FOXG1 (Hou et al., 2020) (Figure 6g,h), and a higher number of cells positive for the muscle marker TNNT2 (Figure 6g).

Taken together, scRNA‐seq, proteomics and immunocytochemistry showed a decrease in neural differentiation and an altered lineage commitment trajectory in AxD cortical organoids, complementing the previous observations of abnormal neurodevelopment in AxD unguided neural organoids and in AxD co‐cultures.

### 3.7 Co‐culture clusters resemble the cell populations in unguided neural organoids and cortical organoids

Given that the astroAxD/neuroC co‐cultures, AxD unguided neural organoids, and AxD cortical organoids all showed impaired differentiation of astrocytes and neurons, we explored the similarity of the cell populations and the effect of the GFAP^R239C^ mutation across the models. We projected the top 10 co‐culture cluster marker genes on the unguided neural and cortical organoid UMAPs (Figure S8A–C) and we also identified marker genes overlapping between co‐culture clusters and all the organoid cell populations (Figure S8D,E).

In the unguided neural organoid dataset, co‐culture clusters including mature ASTRO 2, NEURO 2, and peripheral NEURO 4 shared several marker genes with the organoid populations of astrocytes (*S100B*, *FABP7*, and *GFAP*), neurons (*NNAT* and *SCG2*), and peripheral neurons (*ISL1*, *PRPH,* and *PHOX2B*). Interestingly, immature ASTRO 1 showed similarities not only to the organoid astrocytes (*CD9*, *CRIP2,* and *CLU*), but also to the mesenchymal‐like population (*ELN*, *HSPB6*, *MGP,* and *COL6A2*). We also found that the AxD cluster mapped to the populations of epithelial (*KRT81*, *EPCAM*, *JUP*, *SAT1,* and *ELF3*) and mesothelial cells (*KRT19*, *CDH3*, *SDC4,* and *DSG2*), sharing several genes with the pancreatic‐like acinar cells (*KRT18*, *KRT8*, *DSP*, *FLNB,* and *LAMA5*), which are also of epithelial origin (Ma et al., 2023) (Figure S8B,D).

We also overlapped the astrocyte‐neuron co‐culture clusters and cell populations of cortical organoids and found that the AxD cluster and clusters of less mature ASTRO 1 in co‐cultures mapped to RG (*SLC2A3*, *SLC2A1* and *NGFR*, *CLU*, respectively), oRG glia/astrocytes (*APOE*, *EGFR* in the AxD cluster and *CD9* in both) and to the cell populations that exhibited an aberrant differentiation and were enriched in AxD cortical organoids (*FN1*, *PLEC* and *COL6A2*, *ITGA7*) (Figure S8C,E). Although the ASTRO 2 signature appeared more scattered, since the control cortical organoids largely lacked mature astrocytes, it was enriched in oRG/astrocyte (*COL11A1*, *CRYAB*, *DBI*, and *SIRT2*) and RG populations (*FABP7*, *PTPRZ1*, and *ID4*). Despite the lack of shared marker genes, the mature NEURO 2 signature was detected mostly in neuronal clusters of the control cortical organoids, and the signature of immature NEURO 1 was localized to AxD‐enriched RG and mesenchymal‐like and muscle cells in organoids.

Overall, these data indicate an aberrant differentiation of astrocytes and neurons in three different AxD models derived from AxD patient iPS cells carrying the GFAP^R239C^ mutation, potentially pointing to a new unexplored pathophysiological mechanism in AxD patients.

## 4 DISCUSSION

AxD is a devastating disorder caused by mutations in *GFAP*, with no effective treatment available. Apart from the recently introduced rat model (Hagemann et al., 2021), other animal models do not fully recapitulate AxD pathology or require simultaneous presence of the AxD mutation and an overexpression of normal GFAP (Hagemann, 2022). Patient iPS cell‐derived models allow to study AxD pathogenesis in a human model system. Previously, these models were shown to recapitulate some neuropathological hallmarks of AxD including RFs (Battaglia et al., 2019; Canals et al., 2018; Jones et al., 2018; Kondo et al., 2016; Li, Tian, et al., 2018), revealed abnormal organelle morphology and distribution (Jones et al., 2018), shed some light on the impact of AxD astrocytes on oligodendrocytes (Li, Tian, et al., 2018), and suggested a role of GFAP hyperphosphorylation in AxD pathology (Battaglia et al., 2019). In this study, we used an existing patient‐derived iPS cell line carrying the GFAP^R239C^ mutation and its respective isogenic control cell line (Battaglia et al., 2019) to generate an astrocyte‐neuron co‐culture system that combines isogenically corrected neurons with AxD astrocytes. We also generated unguided neural organoids and cortical organoids from the same AxD and corrected control iPS cell line. In all three systems, we observed a distinct differentiation phenotype pointing to the effect of the GFAP^R239C^ mutation on neural development.

Astrocyte intermediate filaments positively contribute to the ability of astrocytes to handle various stresses (Pekny & Lane, 2007; Ridge et al., 2022), including mechanical, ischemic, and hypoxic stress (De Pablo et al., 2013, Ding et al., 1998, Li et al., 2008, Lundkvist et al., 2004, Nawashiro et al., 1998, Verardo et al., 2008, Wunderlich et al., 2015). Here, we report signs of an increased stress response of human iPS cell‐derived AxD co‐cultures as well as their increased sensitivity to OGD‐induced stress. DEGs in astroAxD/neuroC co‐cultures showed increased stress response as indicated by upregulation of metallothioneins (*MT2A* and *MT1X*). This supports previous observations of increased oxidative stress and activation of stress response pathways in AxD astrocytes (Hagemann et al., 2005; Heaven et al., 2022; Sosunov et al., 2018; Viedma‐Poyatos et al., 2022; Wang et al., 2011), including the upregulation of metallothioneins (Hagemann et al., 2005). The exposure of astrocyte‐neuron co‐cultures to a mild OGD challenge followed by a recovery period identified an increased susceptibility to stress in the astroAxD/neuroC co‐cultures. This is in agreement with the previous reports showing that AxD astrocytes exhibit increased sensitivity to specific stresses (Cho & Messing, 2009; Viedma‐Poyatos et al., 2022). One possible link is the lack of PDGF‐C to PDGFRα signaling potential in astroAxD/neuroC, but not in astroC/neuroC, co‐cultures after exposure to OGD, since PDGF‐C was previously shown to be induced by the stress triggered by radiation injury (Miyata et al., 2014). OGD in astroAxD/neuroC co‐cultures induced signaling potential through several pathways that were absent in corrected control co‐cultures exposed to OGD, and this could reflect maladaptive effects: SPP1 and NRG were reported to have multiple detrimental roles in the CNS and other tissues, respectively (Basak et al., 2023; Cappellano et al., 2021; Schramm et al., 2022). Exposure to various stresses during in vitro differentiation or prenatal development is known to result in a differentiation/developmental delay, and consequently, it is possible that the increased sensitivity of the AxD cells to stress is a driving factor behind the impaired development seen in AxD co‐cultures and AxD neural organoids.

Using scRNA‐seq, we found a population of less differentiated cells in astrocyte‐neuron co‐cultures containing AxD astrocytes. These cells, which we termed the AxD cluster, were characterized by genes expressed by epithelia (Dong et al., 2018). Importantly, in co‐cultures containing AxD astrocytes, less differentiated cells were also identified within the populations of astrocytes and neurons, and we observed downregulation of astrocyte‐specific marker genes (e.g., *GFAP*, *S100B*). The assessment of morphological parameters of GFAP^+^ cells in co‐cultures showed more RG‐like morphology of AxD astrocytes compared to controls. Unguided neural organoids are well suited for investigating differentiation, as they allow a variety of cell types to develop and self‐organize in a 3D environment, and in that sense mimic the in vivo development (Lancaster & Knoblich, 2014; Ormel et al., 2018; Verkerke et al., 2024). In 165 days old AxD unguided neural organoids, astrocytes were almost absent, and neurogenesis was reduced. Our data suggest a neural lineage commitment defect in AxD unguided neural organoids that resulted in an aberrant differentiation, generating epithelial, mesoderm‐derived, or pancreatic acinar cells. Interestingly, Hagemann et al. (2005) reported in a mouse model of AxD, a downregulation of genes related to neuronal development and function, and this might reflect a neuronal loss or impaired neurodevelopment in AxD. Another, more advanced study, showed that AxD mice have reduced proliferation of hippocampal neural progenitor cells, decreased adult neurogenesis, RG with atypical morphology and an increased fraction of undifferentiated neural cells, possibly due to dysregulation of pathways regulating renewal of neural stem cells and neural differentiation (Notch, WNT, Hedgehog, and TGFβ) (Hagemann et al., 2013).

Here we show a dysregulation of some of the same pathways in astroAxD/neuroC co‐cultures and in AxD cortical organoids using CellChat analysis. Signaling pathways such as Notch, WNT, FGF, or EGF, are known to regulate both CNS and pancreatic development (Alkailani et al., 2022; Gonçalves et al., 2021; Lampada & Taylor, 2023; Li et al., 2015; Napolitano et al., 2023; Tomé et al., 2023; Zhang et al., 2023). Therefore, an imbalance of these pathways in AxD organoids may have resulted in the increased presence of cell populations other than those of neuroectodermal origin. These pathways are regulated by the proteasome system (Baloghova et al., 2019; Dutta et al., 2022; Gao et al., 2014; Hsia et al., 2015; Imamura et al., 2013), which was previously shown to be defective in AxD due to the presence of GFAP aggregates (Tang et al., 2010).

Cortical organoids were generated using dual SMAD inhibition of the TGFβ and BMP pathways. Interestingly, this did not rescue the differentiation phenotype seen in the AxD unguided neural organoids. The AxD cortical organoids showed impaired neural differentiation with mesoderm‐derived cells dominating over neural cell types. The overrepresentation of RG and neurons lacking *FOXG1* expression, and severe depletion of astrocytes and oligodendrocyte precursors suggest a failure of differentiation into the cortical lineage and impaired gliogenesis. Mass spectrometry analysis of AxD and control cortical organoids revealed a positive correlation between DEGs and DEPs in AxD cortical organoids compared to controls. Proteomics data showed lower levels of the astrocyte marker GFAP, and neuronal proteins α‐internexin, NF‐L, and NF‐M in AxD organoids, corroborating the transcriptomics and immunocytochemical data, and implying impaired neural differentiation in AxD organoids. We observed downregulation of the AQP4 water channel in AxD cortical organoids at the level of protein as well as mRNA, which supports the recently reported finding of a reduced expression of AQP4 in different regions of the CNS in the AxD rat model (Hagemann et al., 2021). The myogenic markers desmin and nestin were upregulated in AxD organoids, as were many of the proteins involved in muscle differentiation or markers of muscle cells (e.g., myosins), a finding that further confirms the aberrant differentiation in AxD organoids along the myogenic lineage.

In conclusion, by using a combination of approaches and experimental systems ranging from iPS cell‐derived astrocytes and neurons to iPS cell‐derived neural organoids, we show that the AxD GFAP^R239C^ mutation increases the sensitivity of AxD cells to stress and leads to impaired astrocyte and neuronal differentiation. This finding needs to be validated on iPS cell lines from other AxD patients, and if confirmed, it might indicate that a proportion of individuals carrying the AxD mutations might be lost prenatally as a consequence of impaired neuronal and astrocyte differentiation. It also remains to be seen to what extent this abnormal neural differentiation is reflected in AxD animal models and AxD patients.

## AUTHOR CONTRIBUTIONS

EMH, MP, LV, and HA conceived the study and supervised the experiments, ZM, WD, YDP, OGZ, PA, MK, IC, and CAGHVG and HRV performed the experiments, analyzed and interpreted the data, ZM, WD, YDP, DPS, LV, EMH, and MP prepared the manuscript and figures, and all the authors edited and approved the manuscript.

## CONFLICT OF INTEREST STATEMENT

The authors declare no conflicts of interest.

## Supporting information


**Data S1.** Supporting Information.

**Data S2.** Supporting Information.

## Data Availability

The transcriptomic data have been stored at NCBI's Gene Expression Omnibus (Edgar et al., 2002) under accession number GSE261158. Code used for preprocessing and analysis of the transcriptomic data is available at GitHub repository: https://github.com/LabGenExp/hiPSC-derived_AxD_models. The proteomics data have been deposited at the ProteomeXchange Consortium via the PRIDE partner repository with the data set identifier: PXD048606.
