# Supplementary material for: Aberrant neurodevelopment in human iPS cell‐derived models of Alexander disease
Source: Glia. 2024 Sep 23;73(1):57–79. doi: 10.1002/glia.24618 (PMC11660530; doi:10.1002/glia.24618)
Supplement: Supplementary file 1 — Data S1. Supporting Information. [file GLIA-73-57-s001.docx]

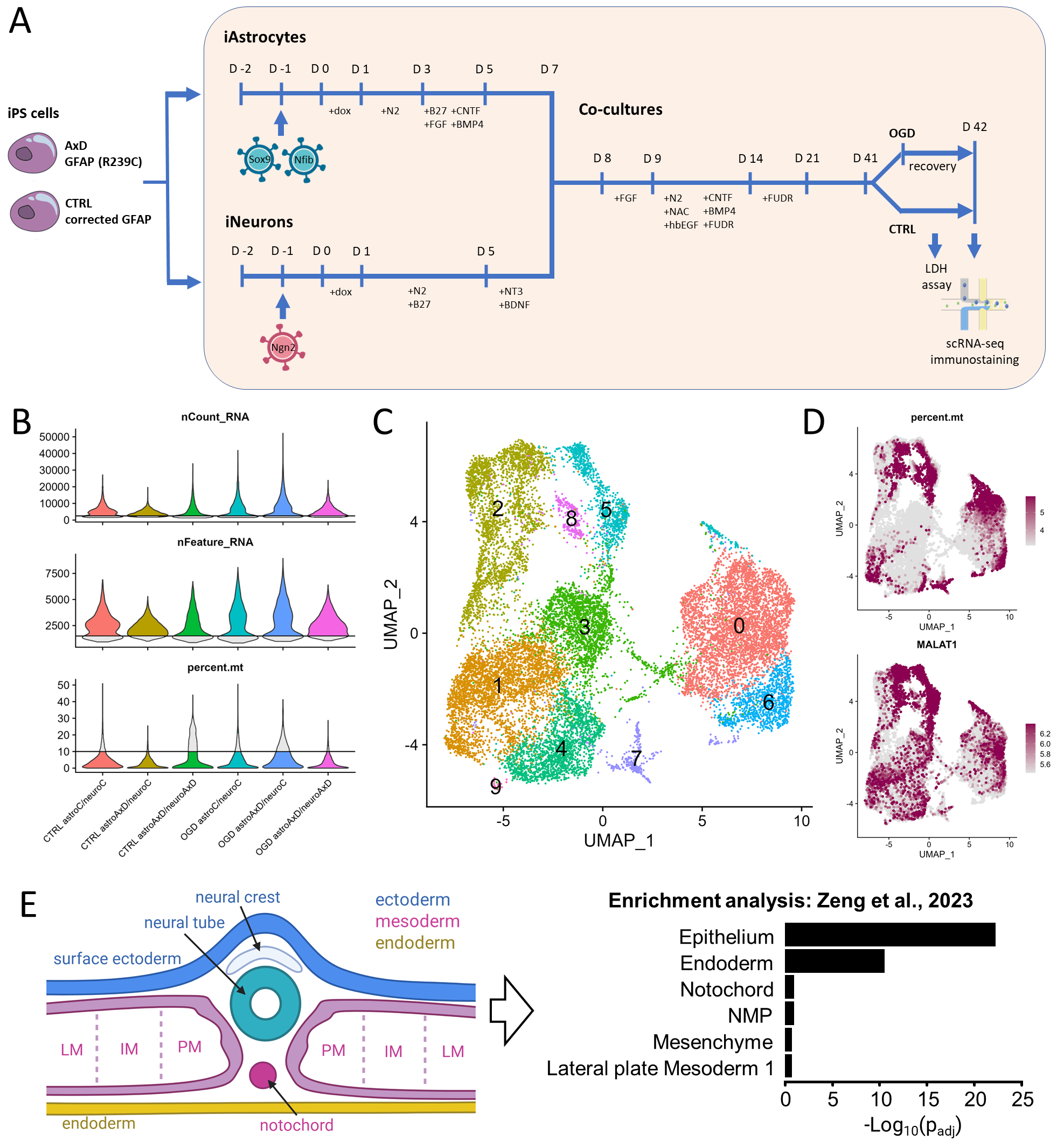


**Supplementary Figure 1:** *Co-culture cultivation scheme and quality control of the co-culture dataset.* **(A)** Timeline of cultivation of iAs and iNs from iPS cells to co-cultures that have been subjected to experiments, including essential media components that changed over time. **(B)** After correction of the dataset with SoupX, the data were filtered based on number of transcripts nCount_RNA > 2500, number of genes nFeature_RNA > 1500, and percentage of mitochondrial genes percent.mt < 10. **(C)** UMAP showing clustering of the filtered data, with clusters 0 and 6 representing induced neurons, clusters 1, 3, 4, 7, and 9 representing induced astrocytes, and clusters 2, 5, and 8 representing the AxD cluster. **(D)** Several clusters were not clearly defined and even after filtering contained high levels of mitochondrial RNA and *MALAT1*. Therefore, they were excluded from further analysis. **(E)** Scheme of early stages of gastrulation and neurodevelopment, when the germ layers and neuroectoderm are specified (left). Surface ectoderm gives rise to epithelia, neural crest to peripheral nervous system, and neural tube to the CNS. Notochord as well as lateral, intermediate and paraxial mesoderm are of mesodermal origin. NMPs are transitional bipotent progenitors generating both neural and mesodermal tissue. This cell type diversity was captured in a single-cell study at 3 PCW by Zeng et al., 2023 and was used as a reference in an overrepresentation analysis of the AxD cluster markers, to clarify their identity (right; p-values adjusted by FDR correction, p_adj_ < 0.2 are shown). **Abbreviations:** astroC/neuroC – corrected co-cultures, astroAxD/neuroC – co-cultures with AxD astrocytes and corrected neurons, astroAxD/neuroAxD – co-culture with AxD astrocytes and neurons, AxD – Alexander disease, B27 – medium supplement for neural progenitor and stem cell cultivation, CNS – central nervous system, CTRL – control without OGD challenge, dox – doxycycline, FUDR – 5-fluoro-2′-deoxyuridine, GO – Gene Ontology, hbEGF – heparin-binding EGF-like growth factor, iAs – induced astrocytes, IM – intermediate mesoderm, iNs – induced neurons, iPS cells – induced pluripotent stem cells, LM – lateral plate mesoderm, LDH – lactate dehydrogenase, N2 – medium supplement for growth of neurons in culture, NAC – N-acetylcysteine, NMP – neuromesodermal progenitors, NT3 – neurotrophic factor, OGD – oxygen-glucose deprivation, PCW – post-conception weeks, PM – paraxial mesoderm, scRNA-seq – single-cell RNA sequencing.


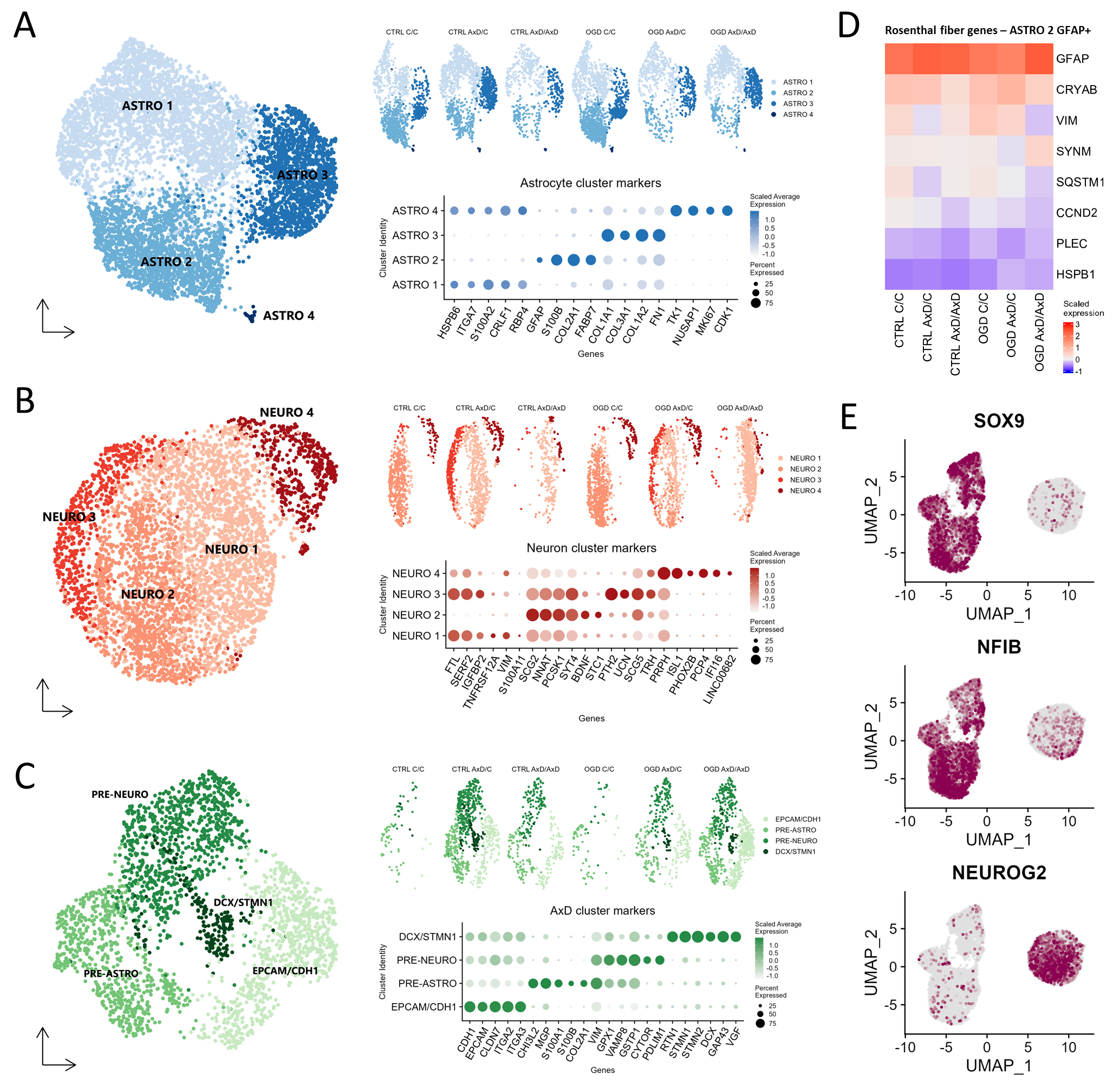


**Supplementary Figure 2:** *Clustering and annotation of populations of iAs, iNs, and the AxD cluster.* **(A-C)** UMAP plots (left) showing clusters of iAs **(A)**, iNs **(B)**, and the AxD cluster **(C)**. Variable representation of individual clusters across samples is shown in split UMAP plots (top right). The clusters have been annotated based on marker genes (bottom right). Wilcoxon test with Bonferroni correction were applied to identify marker genes. **(D)** Scaled average expression of genes associated with Rosenthal fibers in ASTRO 2 cells expressing *GFAP* (*GFAP* counts > 0). **(E)** Expression of transcription factors used for induction of astrocytes and neurons shows that the AxD clustercluster are less differentiated astrocytes rather than neurons. **Abbreviations:** astroC/neuroC, C/C – corrected co-cultures, astroAxD/neuroC, AxD/C – co-cultures with AxD astrocytes and corrected neurons, astroAxD/neuroAxD, AxD/AxD – co-culture with AxD astrocytes and neurons, AxD – Alexander disease, CTRL – control without OGD challenge, iAs – induced astrocytes, iNs – induced neurons, OGD – oxygen-glucose deprivation.


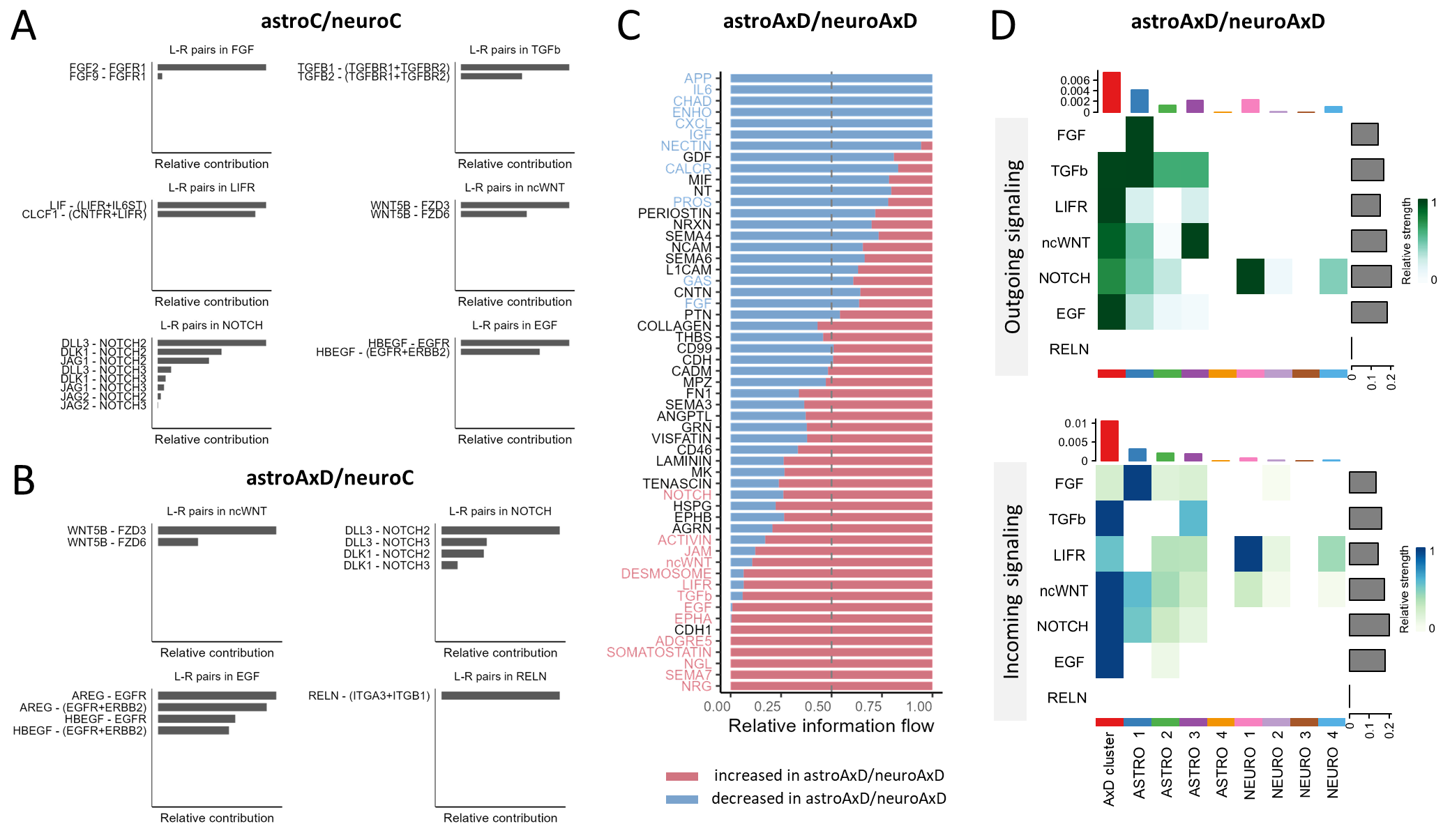


**Supplementary Figure 3:** *Additional data from CellChat analysis of control co-cultures.* **(A)** Specific ligand-receptor pairs of selected pathways and their relative contribution to the signaling in astroC/neuroC co-cultures. **(B)** Specific ligand-receptor pairs of selected pathways and their relative contribution to the signaling in astroAxD/neuroC co-cultures. **(C)** Information flow chart showing pathways detected in astroC/neuroC and astroAxD/neuroAxD co-cultures. Significantly dysregulated pathways (paired Wilcoxon test, p-val < 0.05) are highlighted in colors (red – increase in astroAxD/neuroAxD, blue – decrease in astroAxD/neuroAxD compared to astroC/neuroC). **(D)** Heatmap of astroAxD/neuroAxD CellChat results divided to outgoing (ligands) and incoming (receptors) signaling patterns shows selected neurodevelopmental and astrogenesis-related pathways that were changed in astroAxD/neuroC compared to astroC/neuroC co-cultures. **Abbreviations:** astroC/neuroC – corrected co-cultures, astroAxD/neuroC – co-cultures with AxD astrocytes and corrected neurons, astroAxD/neuroAxD – co-culture with AxD astrocytes and neurons, AxD – Alexander disease, L-R pairs – ligand-receptor pairs.


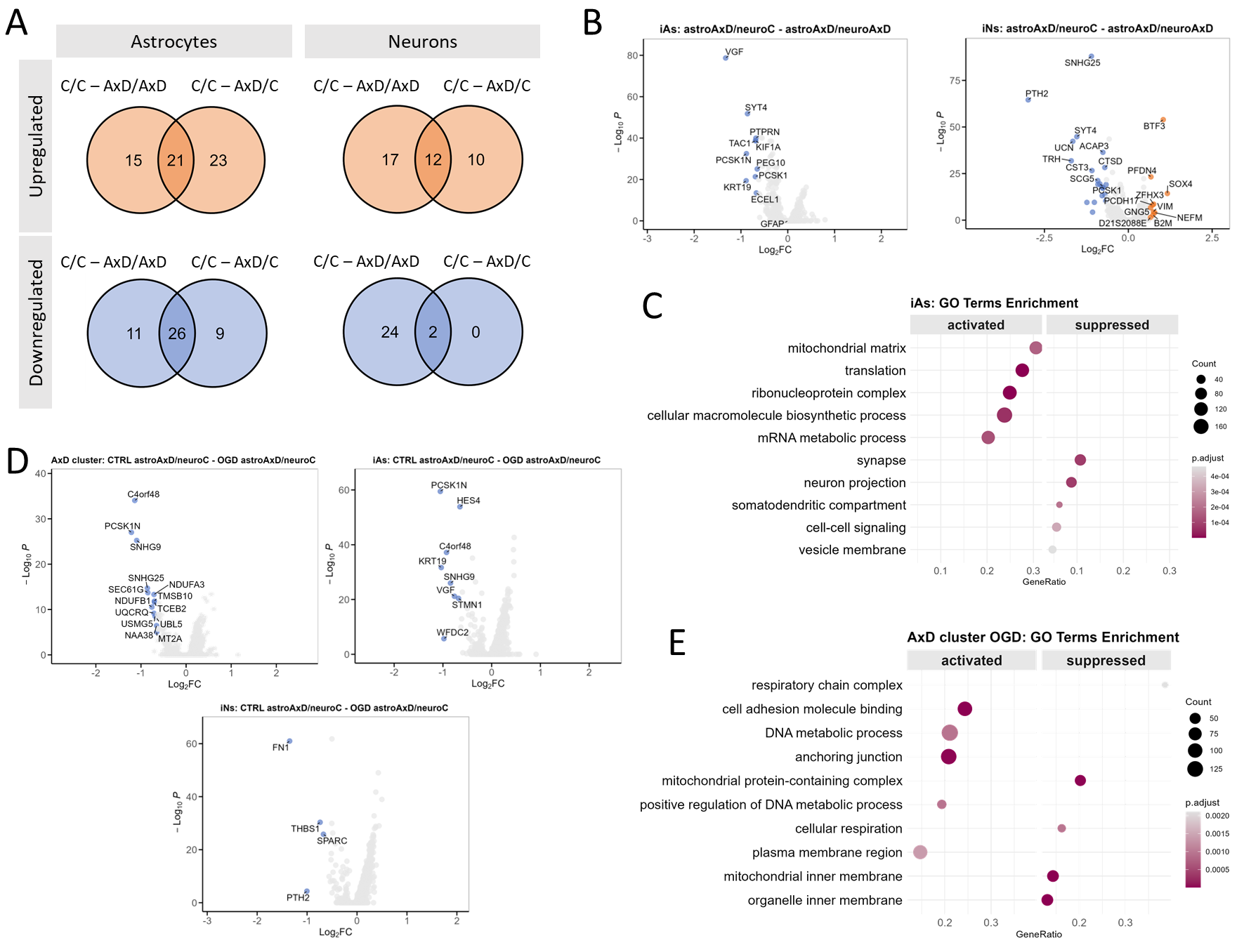


**Supplementary Figure 4:** *Additional results of differential expression analysis of the AxD cluster, iAs, and iNs without OGD and after OGD.* **(A)** Venn diagrams show numbers of dysregulated genes (orange – upregulated, blue – downregulated) in iAs and iNs without OGD that are shared and unique between the comparisons astroC/neuroC – astroAxD/neuroAxD and astroC/neuroC – astroAxD/neuroC. **(B)** Volcano plot showing DEGs between astroAxD/neuroC and astroAxD/neuroAxD astrocytes and neurons (|log_2_FC| > 0.65, p_adj_ < 0.05). **(C)** Top results of GO GSEA of DEGs from the comparison of astroAxD/neuroC and astroAxD/neuroAxD astrocytes (p_adj_ < 0.05; Benjamini-Hochberg correction for multiple comparisons). **(D)** Volcano plots with DEGs identified in the AxD cluster, iAs, and iNs from astroAxD/neuroC co-cultures after OGD compared with control without OGD (|log_2_FC| > 0.65, p_adj_ < 0.05). **(E)** Top results of GO GSEA of the AxD clusterDEGs in astroAxD/neuroC co-cultures after OGD (p_adj_ < 0.05; Benjamini-Hochberg correction for multiple comparisons). DEGs were determined with t-test and Bonferroni correction. **Abbreviations:** astroC/neuroC, C/C – corrected co-cultures, astroAxD/neuroC, AxD/C – co-cultures with corrected neurons and AxD astrocytes, astroAxD/neuroAxD, AxD/AxD – co-cultures with AxD neurons and AxD astrocytes, AxD – Alexander disease, DEGs – differentially expressed genes, GO – Gene Ontology, iAs – induced astrocytes, GSEA – Gene Set Enrichment Analysis, iNs – induced neurons, log_2_FC – log_2_ fold change, OGD – oxygen-glucose deprivation, p_adj_ – adjusted p-value.


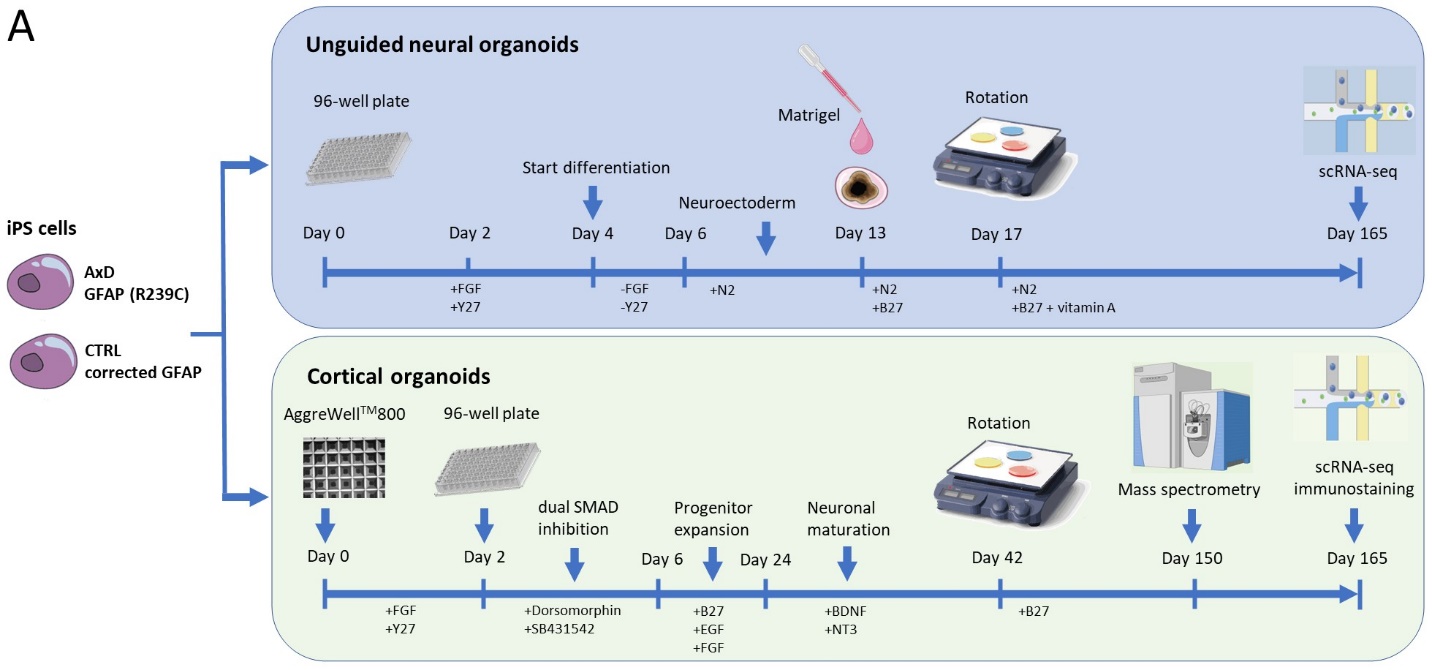


**Supplementary Figure 5:** *A scheme of organoid cultivation timeline and experiments.* **(A)** The scheme summarizes the cultivation process of unguided neural as well as cortical organoids including the essential media components that changed over time. Days when the samples were harvested for experiments are also marked in the timeline. **Abbreviations:** AxD – Alexander disease, B27 – medium supplement for neural progenitor and stem cell cultivation (without vitamin A) or for neuronal growth (with vitamin A), CTRL – corrected control, ECM – extracellular matrix, iPS cells – induced pluripotent stem cells, N2 – medium supplement for growth of neurons in culture, NT-3 – neurotrophic factor, scRNA-seq – single-cell RNA sequencing, Y27 – Y27632 ROCK inhibitor.


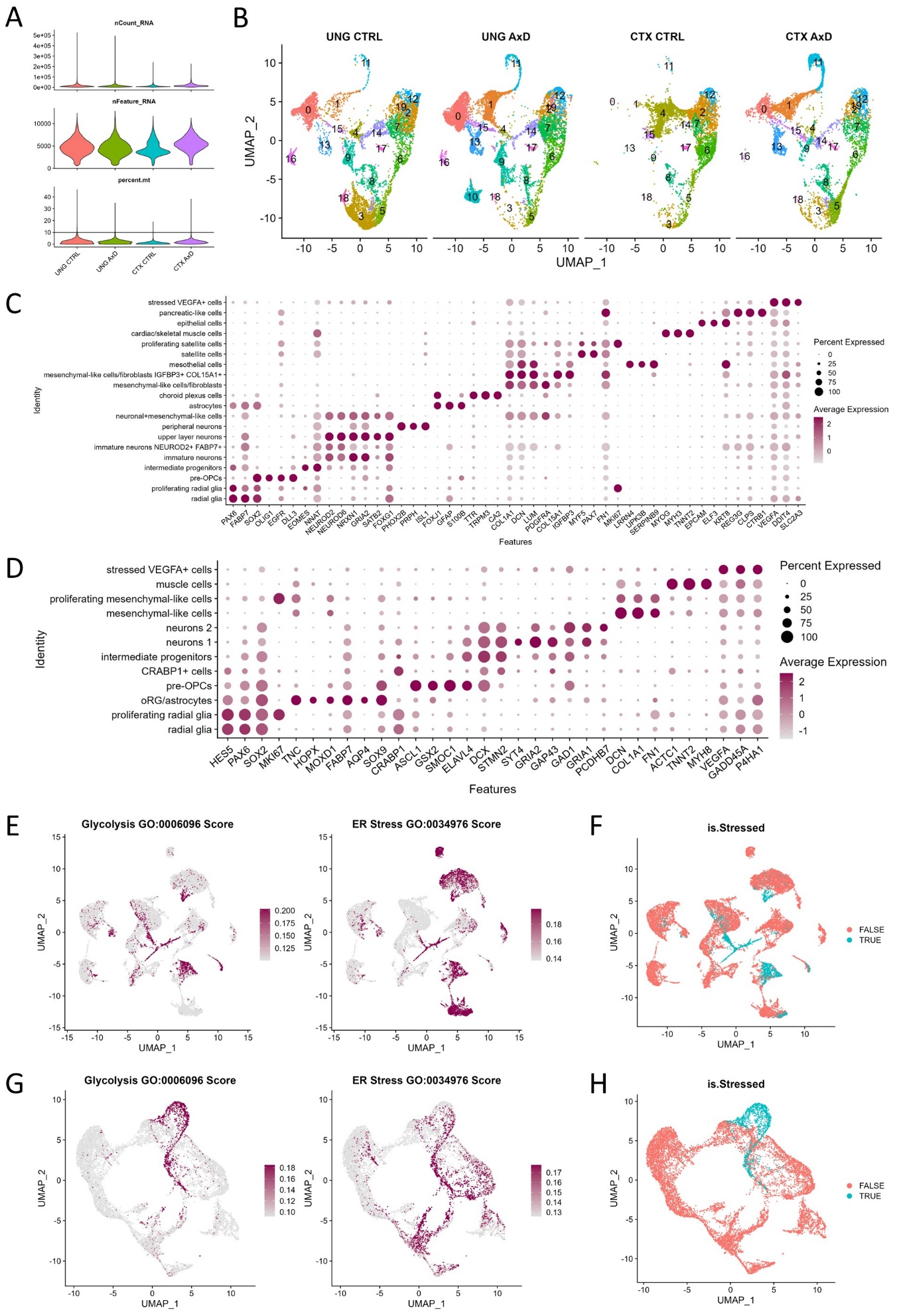


**Supplementary Figure 6:** *Quality control and annotation of organoid dataset.* **(A)** Violin plots showing distribution of number of transcripts (nCount_RNA), number of genes (nFeature_RNA), and percentage of mitochondrial genes (percent.mt) across 165D-old organoid samples. Cells with percent.mt > 10 were filtered out of the dataset. **(B)** UMAP showing the initial clustering of unguided neural and cortical organoid data together, split by sample. As expected, cluster composition of CTX CTRL differed from UNG CTRL. Therefore, unguided and cortical data were analyzed separately. **(C)** Dotplot showing marker genes used for annotation of clusters in unguided organoid dataset. **(D)** Dotplot showing marker genes used for annotation of clusters in cortical organoid dataset. Marker genes in C and D were calculated using Wilcoxon test with Bonferroni correction. **(E, G)** Scoring of GO terms associated with cell stress in unguided and cortical datasets. **(F, H)** Stressed cells in the unguided and cortical datasets as identified by the Gruffi algorithm; True – cells classified as stressed, False – cells classified as not stressed. **Abbreviations:** AxD – Alexander disease, CTRL – corrected control, CTX – cortical organoids, GO – Gene Ontology, pre-OPCs – pre-oligodendrocyte progenitor cells, UNG – unguided neural organoids.


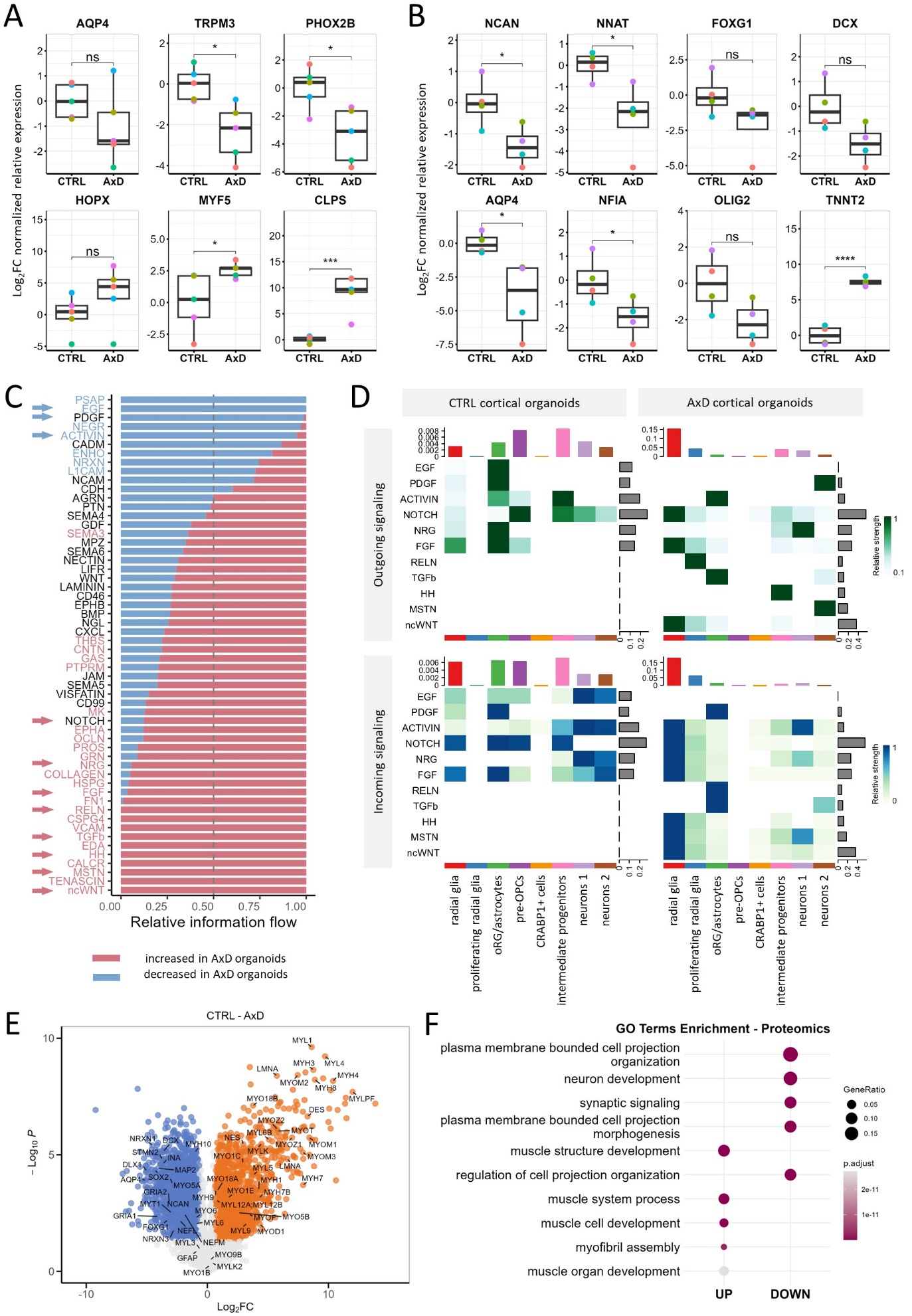


**Supplementary Figure 7:** *Validation of scRNA-seq data from unguided neural and cortical organoids by qPCR, CellChat analysis and mass spectrometry of cortical organoids.* **(A, B)** Log_2_FC normalized relative expression (2^-ΔΔCq^) of selected genes in 165D-old unguided **(A)** and cortical **(B)** organoids measured by RT-qPCR. 2^-ΔΔCq^ values are relative to average ΔCq of controls. Each data point represents one batch (distinguished in colors), and t-test (all but *FOXG1*) and Wilcoxon test (*FOXG1*) were used for statistical comparison; ns – not significant, *: p-val ≤ 0.05, ** p-val ≤ 0.01, *** p-val ≤ 0.001, **** p-val ≤ 0.0001. Shapiro-Wilk test was used to assess normal distribution of the qPCR data. *SDHA*, *TBP*, and *RPII* in unguided and *GAPDH, β-Actin, TBP, SDHA, and RPII* in cortical data were used as reference genes. **(C)** Information flow chart showing pathways upregulated and downregulated in AxD organoids compared with controls. Selected development-related pathways are highlighted with arrows (paired Wilcoxon test, p-val < 0.05). **(D)** The selected pathways in a heatmap showing outgoing and incoming signaling across cell populations. **(E)** Volcano plot of DEPs (|log_2_FC| > 0.65 and p_adj_ < 0.05; t-test with FDR) in 150D-old organoids as identified by mass spectrometry. Myosins, neuronal proteins, and other selected proteins are labeled. **(F)** Top results of GO (biological processes) overrepresentation analysis of DEPs, with p_adj_ < 0.1 (FDR). **Abbreviations:** AxD – Alexander disease, CTRL – corrected control, DEPs – differentially expressed proteins, FDR – false discovery rate, GO – Gene Ontology, log_2_FC – log_2_ fold change, p_adj_ – adjusted p-value, SEM – standard error of the mean.


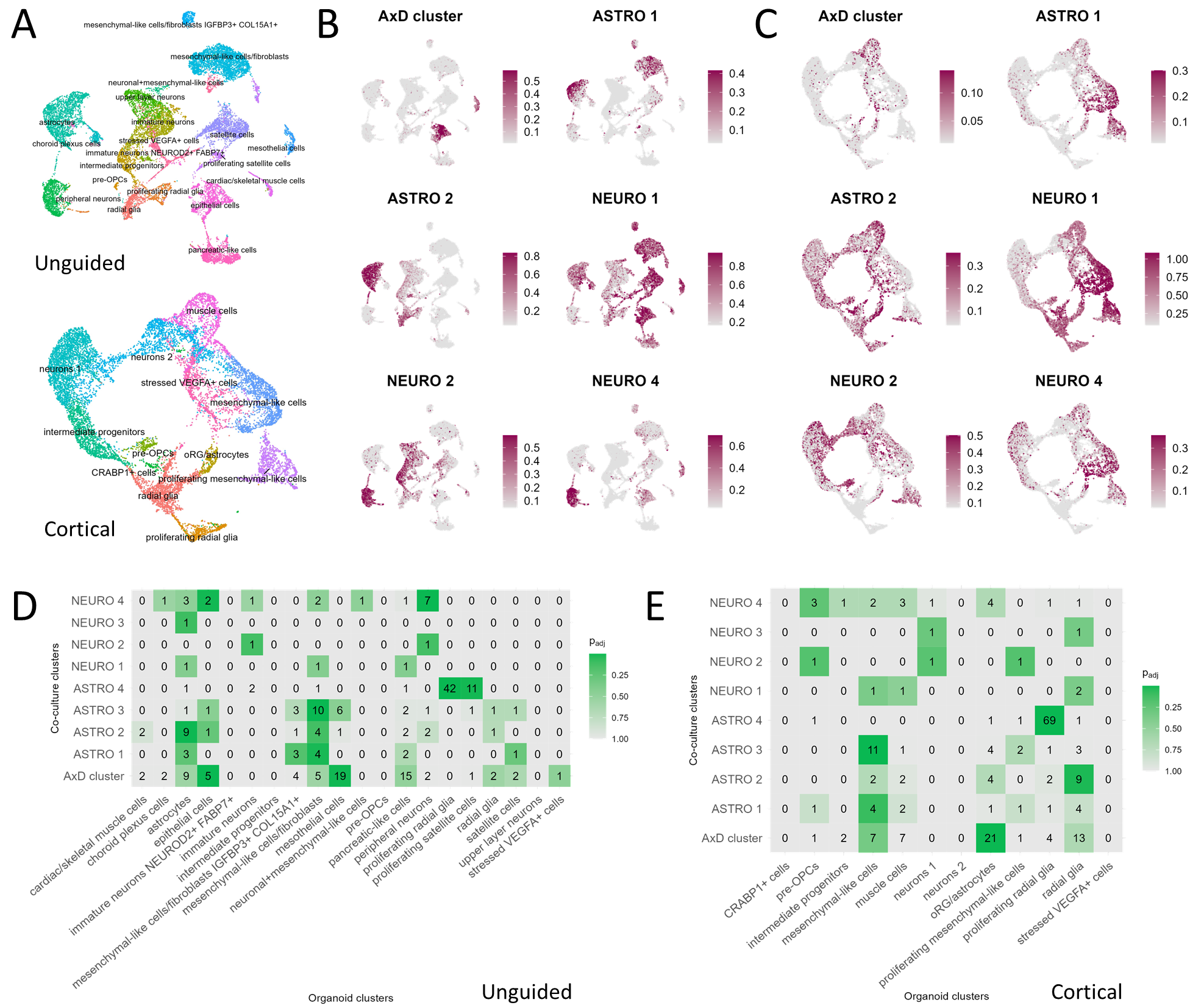


**Supplementary Figure 8:** *Overlap of co-culture data with unguided neural and cortical organoid data.* **(A)** UMAPs of unguided neural and cortical organoids with cluster labels. **(B, C)** Top 10 markers of co-culture clusters were projected onto organoid UMAPs (unguided in **(B)** and cortical in **(C)**) as a score (*Seurat::AddModuleScore()*). **(D, E)** Heatmaps showing number of marker genes shared between co-culture and organoid clusters (unguided in **(D)** and cortical in **(E)**). Color scale corresponds to the level of significance of this overlap determined by adjusted p-value (p_adj_ determined by Benjamini-Hochberg correction for multiple comparisons; per row corresponding to one overrepresentation analysis).


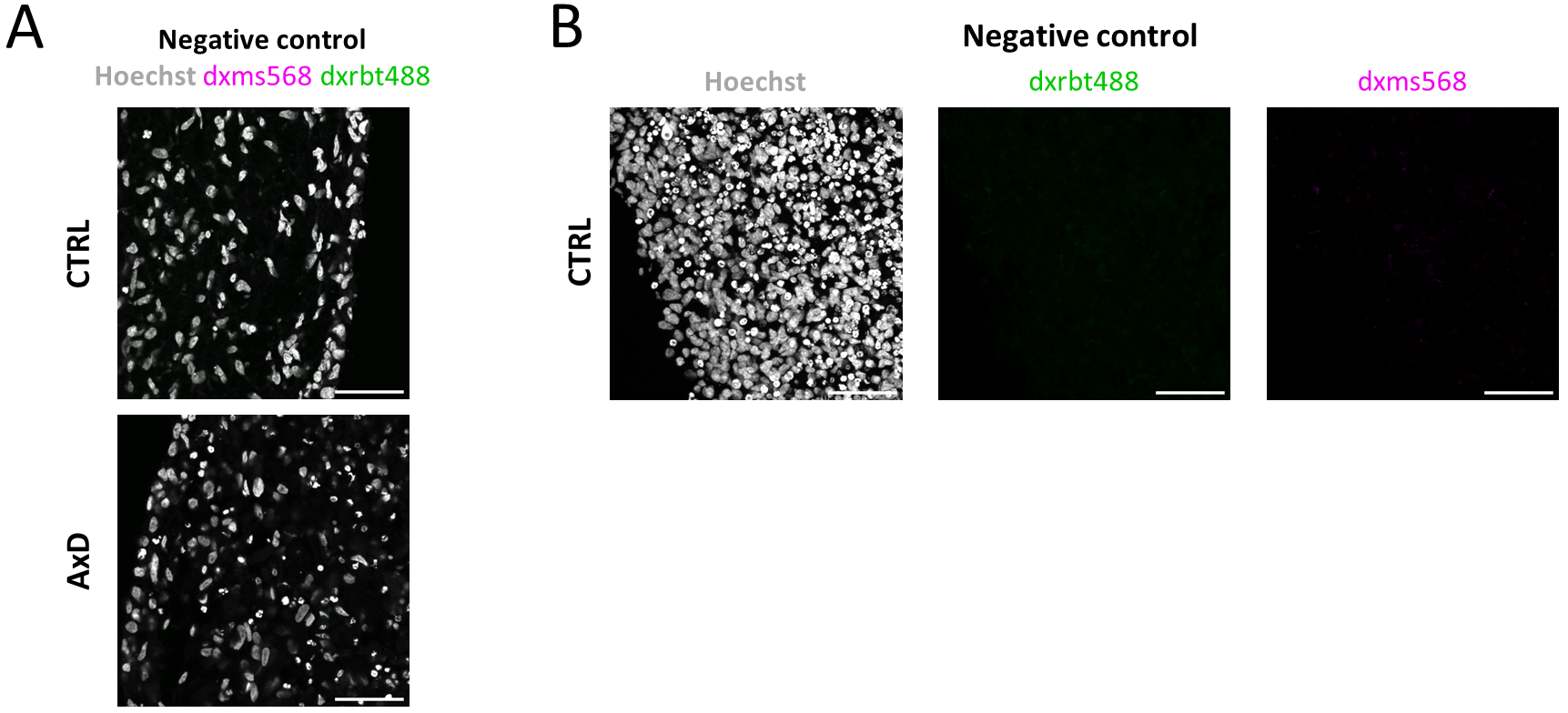


**Supplementary Figure 9:** *Negative controls for immunocytochemistry of unguided and cortical organoids.* **(A)** Negative control for unguided organoids, merged nuclei Hoechst staining with secondary antibodies only (dxms568, dxrbt488). Scale bar, 50 μm. **(B)** Negative control for cortical organoids, nuclei Hoechst staining and secondary antibodies only (dxms568, dxrbt488). Scale bar, 50 μm. **Abbreviations:** AxD – Alexander disease, CTRL – control, dxms568 – donkey anti-mouse, Alexa Fluor 568*,* dxrbt488 – donkey anti-rabbit, Alexa Fluor 488.

**Supplementary table 1:** *List of qPCR primers used for organoid experiments*

| **Gene** | **Forward (5’🡪3’)** | **Reverse (5’🡪3’)** |
| --- | --- | --- |
| *GAPDH* | TGCACCACCAACTGCTTAGC | GGCATGGACTGTGGTCATGA |
| *B-actin* | GTGGACATCCGCAAAGACCT | TCTGCATCCTGTCGGCAAT |
| *TBP* | CCACAGCTCTTCCACTCACA | GCGGTACAATCCCAGAACTC |
| *SDHA* | GAAGCCCTTTGAGGAGCACT | GTTTTGTCGATCACGGGTCT |
| *RPII* | GCACCACGTCCAATGACAT | GTGCGGCTGCTTCCATAA |
| *AQP4* | AAACGGACTGATGTCACTGGC | AACTGCAGGTCCAAAGGATCG |
| *TRPM3* | GGAAAGGGCTCATCAAAGCAG | CCAACATGACGAATAACACCTGT |
| *PHOX2B* | CTTGGGGCTCTTCTTCAGGG | ACAGTCTGAGCAAGTCGTCG |
| *HOPX* | GAGACCCAGGGTAGTGATTTGA | AAAAGTAATCGAAAGCCAAGCAC |
| *MYF5* | CTGCCAGTTCTCACCTTCTGA | AACTCGTCCCCAAATTCACCC |
| *CLPS* | TGTGGCCTATGCAGCTCCT | CTCACCGTTCTCCAGGTTGA |
| *FOXG1* | GAGCGACGACGTGTTCATC | GCCGTTGTAACTCAAAGTGCTG |
| *DCX* | TTCAAGGGGATTGTGTACGCT | GTCAGACAGAGATCGCGTCAG |
| *NFIA* | ATCCCTCAACAGACACAGTCCT | GAGTTGAGAAGGGGTCAGGTG |
| *OLIG2* | AGGACAAGAAGCAAATGACAG | TCCATGGCGATGTTGAGG |
| *TNNT2* | TTCGACCTGCAGGAGAAGTT | GCGGGTCTTGGAGACTTTCT |

**Supplementary table 2:** *List of antibodies used for co-culture and organoid experiments*

|  | **Antigen** | **Host** | **Manufacturer** | **Catalog No.** | **Dilution** |
| --- | --- | --- | --- | --- | --- |
| Co-cultures | GFAP | Guinea Pig | Synaptic Systems | 173004 | 1:500 |
|  | Vimentin (V9) | Mouse | Dako (Agilent) | M0725 | 1:500 |
|  | MAP2 | Chicken | Abcam | ab5392 | 1:5000 |
|  | Anti-Guinea Pig IgG Alexa Flour 488 | Goat | Invitrogen | A-11073 | 1:500 |
|  | Anti-Mouse IgG Alexa Flour 488 | Donkey | Invitrogen | A-21202 | 1:500 |
|  | Anti-Chicken IgY Alexa Flour 568 | Donkey | Invitrogen | A78950 | 1:500 |
| Organoids | GFAP | Rabbit | Agilent, DAKO | Z0334 | 1:1000 |
|  | Vimentin | Chicken | Merck | AB5603 | 1:1000 |
|  | Neuronatin | Rabbit | Abcam | Ab27266 | 1:500 |
|  | Colipase | Rabbit | Thermo Scientific | PA5-52699 | 1:500 |
|  | SOX9 | Rabbit | Cell Signaling Technologies | 82630S | 1:500 |
|  | Cardiac Troponin T | Mouse | Abcam | ab8295 | 1:500 |
|  | Doublecortin | Mouse | EMD Millipore | MABN707 | 1:500 |
|  | FOXG1 | Rabbit | Abcam | ab18259 | 1:500 |
|  | Neurofilament (Pan axonal) | Mouse | Covance | SMI312 | 1:500 |
|  | Anti-rabbit IgG, Alexa Fluor 488 | Donkey | Jackson Immunoresearch | 711-545-152 | 1:1000 |
|  | Anti-rabbit IgG, Alexa Fluor 568 | Donkey | Abcam | Ab175470 | 1:1000 |
|  | Anti-mouse IgG, Alexa Fluor 568 | Donkey | Life Technologies | A10037 | 1:1000 |
|  | Anti-chicken IgY, Alexa Fluor 488 | Donkey | Jackson Immunoresearch | 703-545-155 | 1:1000 |

**Supplementary Table 3:** *Table summarizing sequencing library preparation and data details for each sample in both scRNA-seq experiments*

| Single-cell library preparation and sequencing details | | | | | | | | | |
| --- | --- | --- | --- | --- | --- | --- | --- | --- | --- |
| Sample name | | | # of preamplification PCR cycles | # of indexing PCR cycles | Total # of reads | % of reads mapped to unique genes | EmptyDrops UMI threshold | # of cells after emptyDrops | # of Cells after QC* |
| Co-cultures | CTRL astroC/neuroC | | 12 | 12 | 121565135 | 59 | 2000 | 3831 | 2074 |
|  | CTRL astroAxD/neuroC | |  |  | 118699737 | 51 |  | 5278 | 3372 |
|  | CTRL astroAxD/neuroAxD | |  |  | 124013330 | 43 |  | 2692 | 1198 |
|  | OGD astroC/neuroC | |  |  | 189057949 | 54 |  | 5187 | 2691 |
|  | OGD astroAxD/neuroC | |  |  | 153729507 | 52 |  | 3864 | 1872 |
|  | OGD astroAxD/neuroAxD | |  |  | 163102826 | 42 |  | 4276 | 2810 |
| 165-D organoids | | UNG CTRL | 8 | 10 | 222466396 | 90 | 1000 | 8227 | 7825 |
|  |  | UNG AxD |  |  | 282231331 | 90 |  | 11321 | 10786 |
|  |  | CTX CTRL |  |  | 217868418 | 90 |  | 5550 | 5329 |
|  |  | CTX AxD |  |  | 242649041 | 90 |  | 7042 | 6717 |
|  | |  |  |  |  |  | * after filtering and quality control (QC) steps | | |
